# The Transition Toward Nitrogen Deprivation in Diatoms Requires Chloroplast Stand-By and Deep Metabolic Reshuffling

**DOI:** 10.3389/fpls.2021.760516

**Published:** 2022-01-18

**Authors:** Matteo Scarsini, Stanislas Thiriet-Rupert, Brigitte Veidl, Florence Mondeguer, Hanhua Hu, Justine Marchand, Benoît Schoefs

**Affiliations:** ^1^Metabolism, Bio-Engineering of Microalgal Molecules and Applications (MIMMA), Mer Molécules Santé, IUML—FR 3473 CNRS, Le Mans University, Le Mans, France; ^2^Institut Pasteur, Genetics of Biofilms Laboratory, Paris, France; ^3^Phycotoxins Laboratory, Institut Français de Recherche pour l'Exploitation de la Mer, Nantes, France; ^4^Key Laboratory of Algal Biology, Chinese Academy of Sciences, Wuhan, China

**Keywords:** carbon metabolism reorientation, stress, biotechnology, lipids, transcriptomics, turbidostat operated photobioreactor, pigments, photosynthesis

## Abstract

Microalgae have adapted to face abiotic stresses by accumulating energy storage molecules such as lipids, which are also of interest to industries. Unfortunately, the impairment in cell division during the accumulation of these molecules constitutes a major bottleneck for the development of efficient microalgae-based biotechnology processes. To address the bottleneck, a multidisciplinary approach was used to study the mechanisms involved in the transition from nitrogen repletion to nitrogen starvation conditions in the marine diatom *Phaeodactylum tricornutum* that was cultured in a turbidostat. Combining data demonstrate that the different steps of nitrogen deficiency clustered together in a single state in which cells are in equilibrium with their environment. The switch between the nitrogen-replete and the nitrogen-deficient equilibrium is driven by intracellular nitrogen availability. The switch induces a major gene expression change, which is reflected in the reorientation of the carbon metabolism toward an energy storage mode while still operating as a metabolic flywheel. Although the photosynthetic activity is reduced, the chloroplast is kept in a stand-by mode allowing a fast resuming upon nitrogen repletion. Altogether, these results contribute to the understanding of the intricate response of diatoms under stress.

## Introduction

Since the industrial revolution, the human population and cities size have risen. To sustain the continuous increase in the energy demand, fossil energies were massively used, becoming progressively limited. The accumulation of greenhouse gasses and pollutants in the atmosphere has serious environmental consequences to a point that alternative and more sustainable sources have to be developed (Moss et al., 2010; Levy and Patz, 2015). Being the only living organisms able to use inorganic carbon to produce organic compounds, photosynthetic organisms constitute an alternative choice. However, the large exploitation of plants for other purposes than food and feed production resulted in a counterproductive competition for agricultural land use, rocking the inflation of food prices (Kazamia and Smith, 2014). Microalgae are another type of photosynthetic organisms that can be grown in open ponds or photobioreactors (*i.e*., farmland areas, thus avoiding the land competition) with the aim to use them as cell factories in production platforms (Gordon et al., 2019). Among microalgae, diatoms represent one of the most abundant groups of marine eukaryotic organisms. Thanks to their unique ability to acclimate to the changing environmental conditions, they have colonized any kind of environment including lichens or mosses (Schoefs et al., 2020). Diatoms have always played a crucial role in the biosphere as they contribute to 20–40% of the oceanic biomass production and approximately 20% of the total carbon fixation (Benoiston et al., 2017; Falciatore et al., 2020). Their metabolic diversity and flexibility make them attractive organisms for a wide range of applications in diverse industrial and commercial fields, including biofuel production, nanotechnological applications, pigments and dietary lipids production (Mimouni et al., 2012).

Despite the tremendous progress in cultivation systems, the use of diatoms as cell factories remains in its infancy. Recent analyses pointed out the high cost of downstream processes and the relative lack of knowledge in diatom biology (Heydarizadeh et al., 2014; Vinayak et al., 2015). The actual view of the cell metabolic network is similar to a marshaling yard which distributes carbon atoms among the different biosynthetic pathways. In non-stressful conditions, the carbon atoms, our train carts, are mostly injected in the pathway producing carbohydrates, used as fuel for the mitochondria to get the energy needed for cell division. Stressful conditions trigger metabolic switches, changing the carbon flow toward the production of energy-dense compounds such as lipids (Sayanova et al., 2017). This metabolic reorientation appears as a default mechanism in diatoms accompanied by a reduction in the division rate (Heydarizadeh et al., 2019). In this study, the marine diatom *Phaeodactylum tricornutum* was selected as the model organism since it presents many advantages, including culture easiness and an annotated genome (Bowler et al., 2008; Butler et al., 2020; Falciatore et al., 2020). Furthermore, the taxon is genetically amenable (Apt et al., 1996; Karas et al., 2015; Serif et al., 2018; Sharma et al., 2018; Fabris et al., 2020; George et al., 2020; Hu and Pan, 2020). The effects of nitrogen starvation on *P. tricornutum* have been widely studied. Unfortunately, the lack of homogeneity in the experimental procedures and parameters does not allow to easily combine the different information to obtain a comprehensive view of the cellular responses from molecular to physiological levels (Supplementary Table 1). Thus, the dynamics and orchestration of the different cellular responses need further clarification. To significantly contribute to this field, a multidisciplinary strategy, including maintaining all parameters, but nitrogen availability, as stable as possible throughout the experiment was adopted: *P. tricornutum* was grown in a highly controlled photobioreactor operated in turbidostat mode, from an unlimited to depleted nitrogen conditions. Molecular, biochemical, and physiological parameters were followed along with the transition. This work shows that the nitrogen-depleted state contains cells in equilibrium with their environment, meaning that the cellular mechanisms no longer evolve qualitatively (change of the cell status), but only quantitatively (no change of the cell status). The qualitative variations, therefore, took place during a transition phase separating the initial equilibrium state (nitrogen repleted) and the final deficient state (N depleted). The transition is characterized by a carbon metabolism reorientation toward energy storage and a reduction of cellular activities, including plastidial ones that are kept on stand-by, waiting for the potential upcoming optimal growing conditions.

## Materials and Methods

### The Photobioreactor

*P. tricornutum* (Pt4) (UTEX 646) was cultivated in an f/2 medium (Guillard and Ryther, 1962) in a photobioreactor (PBR) (FM150, Photon System Instruments, Czech Republic) operated in turbidostat mode (Table 1) using the company software (Version 0.7.14). The PBR was equipped with sensors for light transmission, chlorophyll (Chl) fluorescence, temperature-pH (Mettler Toledo InPro3253SG/120/PT1000), dissolved inorganic carbon (DIC) (Mettler Toledo InPro5000/12/120), and dissolved oxygen (Mettler Toledo InPro6800/12/120). The PBR setup is presented in Supplementary Figure 1. The culture was continuously supplied with 1.2 mL min^−1^ of a mixture of air and CO_2_ (2,000 ppm CO_2_ total). After 5 days of stable growing conditions, the PBR was fed with f/2 medium containing 0.15 mM NaNO_3_. We will refer to the day after the medium was changed as the Day from the Reduction of Nitrogen Supply (DRNS). The pH, temperature, DIC, and O_2_ were continuously monitored. The (Chl) fluorescence yield measurements were carried on in a light-adapted state and in a dark-adapted state of each light-dark cycle (Table 1) (for the definition of the fluorescence parameters, refer to Supplementary Table 2).

**Table 1:** Summary of the cultivation parameters.

**Parameter**	**Value**
Culture volume	1 L
Cell concentration	~3.5 10^6^ cells mL^−1^
Carbon supply	0.24 mL min^−1^
Temperature	21°C
Light	White LED panel, 175 μmol photons s^−1^ m^−2^, light/dark cycle: 23h40/0h20

### Estimation of the Growth Rate

The cultivation in turbidostat mode requires that the culture turbidity remains constant. Turbidity (OD735) was measured at 735 nm to avoid pigment contribution. To maintain the OD735 constant, a peristaltic pump (Photon Systems Instruments PP500) was actioned by the PBR central controller when OD735 increased over a threshold set at 0.151 to maintain a cell density of 4 10^6^ cells^−1^. The medium in excess was pushed in a container and collected as an overflow (14 in Supplementary Figure 1). The daily volume of overflow allows for the estimation of the dilution rate. In fact, the time course of the cell density (x) is a function of the cell division rate (μ_s_) and also of the dilution rate (D) (Equation 1) (Gresham and Hong, 2015).


(1) $$\begin{array}{r} \frac{\text{dx}}{\text{dt}}=\;\mu_{s}\cdot \text{x}-D\cdot \text{x} \end{array}$$

In turbidostat mode, $\frac{\text{dx}}{\text{dt}}$ equals zero and the Equation 1 reduces to Equation 2.


(2) $$\begin{array}{r} \mu_{\text{s}}=D \end{array}$$

The decrease in limiting nutrients induces a decrease in division rate that the automatic dilution compensates accordingly. Thus, the changes in the dilution rate reflect the division rate.

Cells were counted daily using a Neubauer hemacytometer according to Heydarizadeh et al. (2019).

### Sample Harvesting

Samples were sterilely collected (Supplementary Figure 1). For carbohydrate, protein, lipid, and pigment extraction, cells were centrifuged (3,000 × *g*, 10 min, 4°C). Pellets were washed 3 times with a phosphate-buffered solution (PBS) before storage at −80°C. The samples for RNA extraction and metabolite extraction were collected on glass filters (Whatman® glass microfiber filters, Grade GF/A, Merck, Germany) under a gentle vacuum (50 mmHg), immediately frozen in liquid nitrogen, washed 3 times with a PBS solution, and stored at −80°C. Samples for carbon and nitrogen determination were harvested on fiberglass filters under gentle vacuum (50 mmHg), washed 3 times with a PBS solution, and dried at 70°C for 48 h. The sampling volumes and quantities are reported in Supplementary Table 3.

### Elemental Analyses

The nitrate concentration in the medium was determined according to Carvalho et al. (1998). Briefly, the absorbance at 220 nm was measured on a centrifuged culture supernatant (Lambda-25, Perkin-Elmer, USA). A sodium nitrate (NaNO_3_) aqueous solution was used for calibration.

Internal Nitrogen (N-int) and Carbon (C-int) pools were determined using the C7N elemental analyzer (Eager 300, Thermo Scientific, Massachusetts, USA) according to Heydarizadeh et al. (2019).

### RNA Extraction and High Throughput Transcriptome Analysis

The total RNA was extracted from samples with the Spectrum™ Plant Total RNA Kit (Sigma-Aldrich, Missouri, USA). The purified total RNA extracts were processed with an RNAstable® LD kit (Sigma-Aldrich). Paired-end next-generation sequencing has been performed by the Annoroad company (Corporate HQ, Beijing, China) with an Illumina NextSeq 550AR Sequencer. The sequencing results and quality are reported in Supplementary Dataset 1. The raw data were processed with the Trimmomatic tool (version 0.36, Bolger et al., 2014) to remove adapters. A total of 45.9 million high-quality clean reads (96.15% clean reads) were aligned with the Hisat alignment software (version 2.1.0, Kim et al., 2019) on the high-quality reference transcriptome (NCBI assembly accession number: GCF_000150955.2). Expression abundances were quantified using Htseq-count (version 0.11.2, (Anders et al., 2015) and differentially expressed genes (FDR ≤ 0.001; |Log2(FC)| ≥ 1) identified using the R package DESeq (Bioconductor version 3.11, Love et al., 2014). Functional annotations of the transcriptome were carried out using Gene Ontology (GO) terms and enriched with the bioinformatics tool Blast2Go and a manual cross-reference between published data and online public databases (https://protists.ensembl.org/; https://mycocosm.jgi.doe.gov/; https://www.ncbi.nlm.nih.gov/). Heatmaps were generated with an *ad hoc* Python script based on the seaborn repository (gplots version 0.11.0, https://seaborn.pydata.org/index.html). The scripts can be found in the following repository: https://github.com/IlBarbe/SimpleSmallPythonScipts.

### Neutral Lipid Quantification

Neutral lipids were estimated by Nile red fluorescence according to Huang et al. (2019). Pellets were resuspended in dimethyl sulfoxide (DMSO) 25% and stained with 1.5 μL Nile red (Sigma-Aldrich, Saint-Quentin Fallavier, France) solution 1 mg mL^−1^ in DMSO (100%). The 298 K fluorescence at 580 nm was excited at 530 nm (slits 5 nm) (Perkin Elmer LS-55, Villebon sur Yvette, France). DMSO diluted glyceryl trioleate (Sigma-Aldrich T7140-500MG, purity ≥99%) was used for calibration (Supplementary Figure 2).

### Protein Quantification

Protein extraction was performed by mechanical lysis with the FastPrep-24™ 5G Homogenizer (MP Biomed, France). Sample pellets were mixed with glass beads (Merck, USA, 425-600 μm, acid-washed) and 50 μL ultrapure water for mechanical lysis (4 m s^−1^ for 30 s). The sample was chilled in ice for 1 min. After a second lysis cycle, 950 μL of ultrapure water were added to the lysate and homogenized for 30 s. The lysate was centrifuged at 16,000 × *g* for 20 min at 4°C. Protein concentrations were determined using the Bradford method (Bradford, 1976) using a double-beam spectrophotometer (Perkin Elmer Lambda-25). Bovine serum albumin (Sigma-Aldrich, purity ≥ 98%) was used for protein quantification calibration (Supplementary Figure 2).

### Carbohydrate Quantification

Carbohydrate quantification was performed according to a slightly modified Dubois' protocol (Dubois et al., 1956). Briefly, the extraction was performed by mechanical lysis with the FastPrep-24™ 5G Homogenizer (MP Biomed, France). Sample pellets were mixed with glass beads (Merck, 425–600 μm, acid-washed) and 1.5 mL of 0.05 M sulphuric acid-water solution. Extract-containing tubes were incubated at 60°C for 30 min, vortexing every 10 min, then chilled for 10 min on ice. The lysate was centrifuged at 16,000 × *g* for 20 min at 4°C. In glass tubes, 2.5 mL of H_2_SO_4_ (>95%) were added to 1 mL of extract supernatant with the subsequent addition of 250 μL of 3%, phenol-water solution (99%, Merck). The glass tubes incubated in a water bath at 95°C for 30 min and chilled on ice for 10 min. Colorimetric quantification has been performed reading the absorbance at 485 nm using a double-beam spectrophotometer (Perkin Elmer Lambda-25). Glucose (Merck, USA, ≥ 99.5%) was used for total carbohydrate quantification calibration (Supplementary Figure 2).

### Stored Energy Estimation

The energy stored in organic molecules (E_a_) was obtained by converting the different energy storage fractions (*i.e*., neutral lipids, proteins, and carbohydrates) into energetic equivalents using their combustion energy (*i.e.*, Lipids: 39,500 mJ mg^−1^, Proteins: 24,000 mJ mg^−1^, Carbohydrates: 17,500 mJ mg^−1^) (Gnaiger, 1983; Aderemi et al., 2018).

The available energy is thus:


(3) $$\begin{array}{r} {\text{E}}_{\text{a}}\left(\text{mJ}{\;10}^{6}\text{cell}{\text{s}}^{-1}\right)={\text{E}}_{\text{carbohydrates}}\;+\;{\text{E}}_{\text{lipids}}\;+\;{\text{E}}_{\text{proteins}} \end{array}$$

where E_carbohydrates_, E_lipids_, and E_proteins_ were calculated by multiplying the respective combustion energy by the quantity of corresponding macromolecules *per* 10^6^ cells.

### Pigment Quantification

Pigment extraction was performed by mechanical lysis with the FastPrep-24™ 5G Homogenizer (MP Biomed, France) under green ambient light (Schoefs, 2004). Sample pellets were mixed with glass beads (Merck, 425-600 μm, acid-washed) and 100 μL of pure acetone (Merck, ≥ 99.5%) for mechanical lysis (4 ms^−1^ for 30 s). Samples were centrifuged at 16,000 × *g* for 5 min at 4°C and supernatants were collected in a new microtube. Lysis cycles were repeated until the pellets fade to white. Absorbance spectra of the pooled supernatants were recorded between 400 nm and 800 nm (Perkin Elmer Lambda-25). The Chl *a*, Chl c, and total carotenoids concentrations were calculated according to Heydarizadeh et al. (2017) using the following equations:


(4) $$\begin{array}{r} \text{Chl }a\;\left(\text{mg }{\text{L}}^{-1}\right)\;=\\ \frac{\left[11,77\left(DO_{663nm\;}-DO_{750nm\;}\right)-0.32\left(DO_{663nm\;}-DO_{750nm\;}\right)\right]\times v}{l\times V} \end{array}$$

(5) $$\begin{array}{r} \text{Chl c }\left(\text{mg }{\text{L}}^{-1}\right)=\\ \frac{\left[26.27\left(DO_{630nm\;}-DO_{750nm\;}\right)-3.52\left(DO_{663nm\;}-DO_{750nm\;}\right)\right]\times v}{l\times V} \end{array}$$

(6) $$\begin{array}{r} \text{Total carotenoids }\left(\text{mg }{\text{L}}^{-1}\right)=\\ \frac{\left[1000\left(DO_{473nm\;}-DO_{750nm\;}\right)-8.08\;\times \;Chl\;a\;\times l\;-\;Chl\;b\;\times l\right]\times v}{183.4\times l\times V} \end{array}$$

where *v* is the pigment extraction volume, V is the sampling volume, and l is the optical path.

### Metabolomic Analysis

Metabolomic analyses were performed on samples (*i.e.*, CTRL 4, 5, 6, 7, 8 DNRS, and 10/11 DNRS) grouped on single sample. Metabolite extractions were performed according to Courant et al. (2013). Briefly, samples were plunged into 5 mL of a boiling mixture (ethanol-water, 75:25 v/v, 90–95°C) for 1 min, vigorously shaken for 3 s, and re-incubated for 1 min. The extraction process was then quenched by plunging tubes for 3 min in ethanol water solution (75:25 v/v) at −80°C. After filter removal, the tubes were centrifuged (4,500 × *g*, 5 min, 20°C). The supernatants were filtered on precombusted glass wool and collected in glass vials. The organic solvent phase was evaporated using a rotavapor (Rotavapor R-205 Evaporation System, Buchi; heating bath B-490, Buchi, Vacuum pump PC500, Vacuubrand) and lyophilized (Lyophilizer Christ Alpha 1-2 Plus, Christ; Vacuum pump E2M5, Edwards). The lyophilized extract was then resuspended with 1 mL methanol water solution (20:80 v/v) and stored at −80°C prior to filtration (mesh 0.2 μm). Liquid chromatography-high resolution tandem mass spectrometry (LC-HRMS/MS) analyses were performed with an Agilent 1290 Infinity II LC system coupled to a high-resolution time-of-flight mass spectrometer (Q-Tof 6550 iFunnel, Agilent technologies, CA, USA) equipped with a Dual Jet Stream® electrospray ionization source (positive mode). Detailed information on analyses parameters is reported in Supplementary Information 1. The Agilent Mass Hunter Workstation software (version B.07) was used to process the raw MS data, including the extraction of molecular features (MFs), generation of molecular formulas, library searching, and database searching. The identification of the most relevant entities was supported by MS information and searches in the METLIN database (http://metlin.scripps.edu) and in the Dictionary of Marine Natural Products (DMNP) library (Blunt et al., 2008; Wolfender et al., 2015).

The metabolomic analyses resulted in the detection of 4,267 compounds ranging from 0.1 and 2.8 kDa in size. To reduce the number of compounds to elucidate, the change in compound detected quantity as compared to the change in transcript abundance during the time course of the experiment. As a preliminary step, only compounds and transcripts which significantly changed in abundance, as compared to those under controlled conditions, at least at a time point, were retained. After this first screening, each compound pattern has been compared with each gene expression pattern. To be as stringent as possible, only the combinations which displayed a significant direct correlation were included (|slope|=1, from 0.95 to 1.05; *p* < 0.05). The results showed 419 compound-gene combinations which included 115 compounds. The 115 compounds were kept for further analyses and elucidation.

### Statistics

ANOVA was performed using the online freeware (https://acetabulum.dk/anova.html). Principal component analyses (PCA) were performed with a compiled python script exploiting pandas (version 1.2.1), numpy (version 1.19.5), matplotlib (version 3.3.3), and scikit-learn (version 0.24.1) packages. Propagation of uncertainties in data processing was carried out following the mathematical rules (Arras, 1998). Linear and nonlinear fitting analyses were performed with the free version of CurveExpert software available for download (Hyams, D. G., CurveExpert software, http://www.curveexpert.net, 2010).

## Results

**Figure 1:**
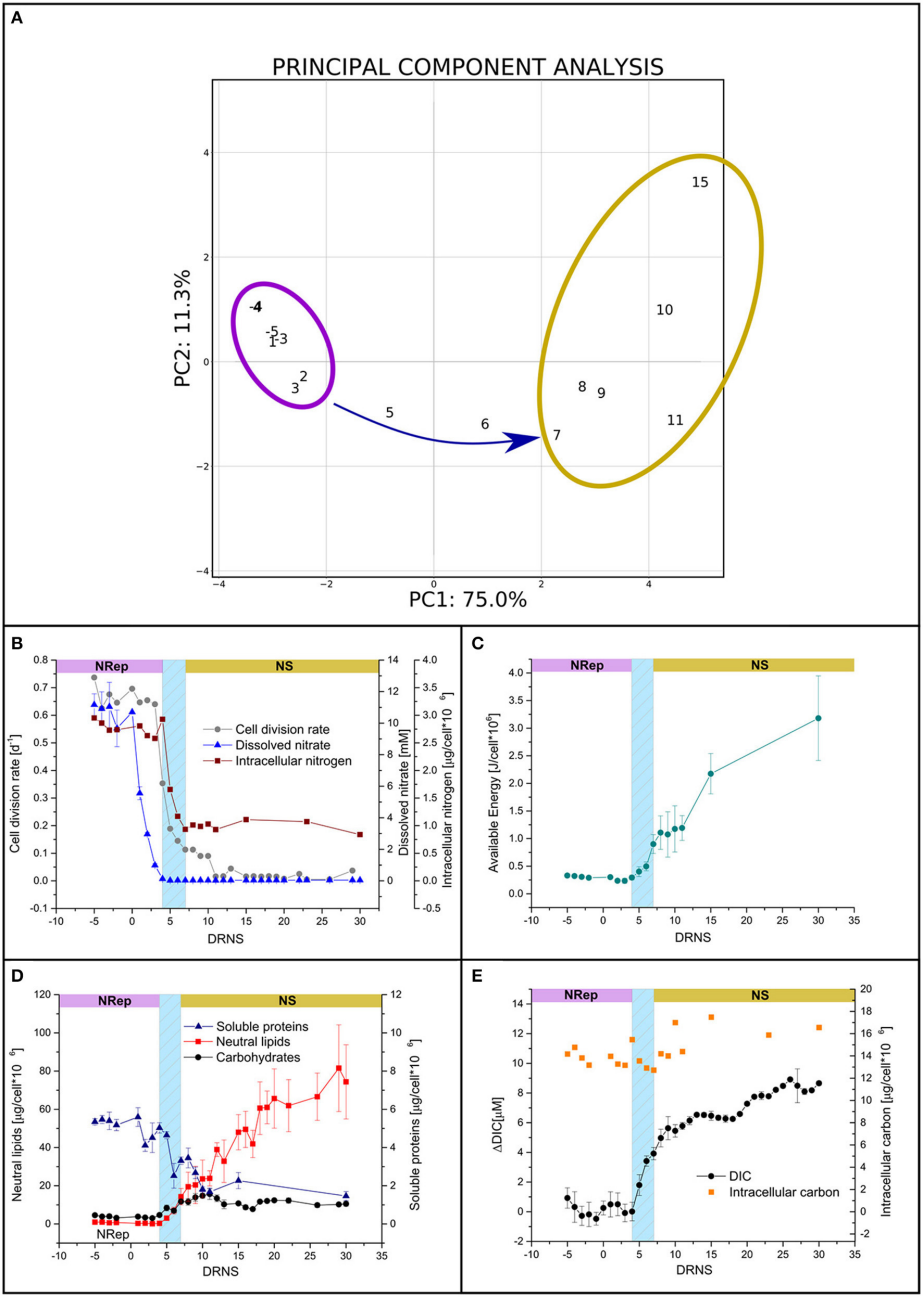
Culture parameters in response to the transition from nitrogen-repleted (NRep) to nitrogen starvation (NS) conditions. **(A)** Principal component analysis (PCA) of the different physiological and biochemical traits. The first two components of the PCA explain 86,3% of the variance, 75% is explained by the first component allowing the outline of two groups: the NRep (Violet) and NS (Yellow) groups and the transition links them together. **(B)** Time-courses of cell density (grey circles), N-int (Red squares), and N-ext (blue triangles). **(C)** Cellular available biochemical energy during the time course of the experiment. Day 0 refers to DNRS. **(D)** Soluble proteins (blue triangles), carbohydrates (black circles), and neutral lipids (red squares) cellular quota. **(E)** DIC and intracellular carbon.

*P. tricornutum* was cultivated in a PBR, operated in turbidostatic mode, and exposed to a 67-fold reduction in nitrogen availability. In order to identify the potential different steps of this transition, a PCA was performed with the biochemical and physiological traits (*i.e.*, DIC, neutral lipid, carbohydrate, protein, total pigment, N-int and C-int cellular quotas, Fv/Fm, qN, qP, and rETR). The first two components explained 86% of the sample variance (Figure 1A). Two groups can be defined: (i) a group containing all the time points from −2 to 4 DRNS that will be referred to as Nitrogen-Repleted (Nrep) condition, and (ii) a second group, from 7 to 15 DRNS that will be referred to as Nitrogen-Starved (NS) condition (Table 2). The two states are linked by a succession of transitory states through days 5 and 6, forming the transition phase. These two timepoints will be considered separately due to the importance of underlining the differences during the transition. The culture entered the transition phase, not at the onset of the DRNS but when the minimum N-ext availability is reached at 5 DNRS (0.03 mM ${\text{NO}}_{3}^{-}$ corresponding to 0.007 μmol ${\text{NO}}_{3}^{-}$10^−6^ cells) (Figure 1B). However, the transition between NRep and NS is rather led by N-int variations which is hereby considered as the key factor. Biochemical and physiological traits during the time course of the experiment were therefore plotted against the change in N-int. The plots, reported in Supplementary Figure 3, confirmed the information given by the performed PCA in which two groups are underlined with the transition in between. From the DRNS, N-ext decreased from 10 mM (NRep) to an average of 0.03 mM (NS) (Figure 1B; Tables 1, 2). With a 4-day delay, the N-int dropped by 64%. Since culture entered the transition phase when minimum N-ext is reached and the NS phase when minimum N-int is reached (Table 2), N can be thus considered as the phase transition governing factor. The cell division rate decreased with timing and kinetic very close to the N-int (Figure 1B).

**Table 2:** Summarizing table presenting the culture parameters during the NRep and NS phases.

**Biochemical and Physiological trait**	**NRep**	**NS**
Dissolved inorganic carbon availability variation [μM]	≃ 4	From 4 to 8
Extracellular inorganic nitrogen availability [mM of ${\text{NO}}_{3}^{-}$]	From 10 to 0.13	≃0.038
Neutral lipid cell quota [μg/10^6^ cells]	≃ 0.5	From 14 to 80
Carbohydrate cell quota [μg/10^6^ cells]	≃ 3.8	≃12
Soluble protein cell quota [μg/10^6^ cells]	≃ 5.08	≃ 2.37
Average pigment cell quota [μg/10^6^ cells]	Chl *a* = 0.18 Chl c *=* 0.024 Car = 19.12	Chl *a =* 0.06 Chl c = 0.016 Car = 7.08
Nitrogen cell quota [μg/10^6^ cells]	≃ 2.78	≃ 0.99
Carbon cell quota [μg/10^6^ cells]	≃ 13.99	≃ 14.86

**Figure 2:**
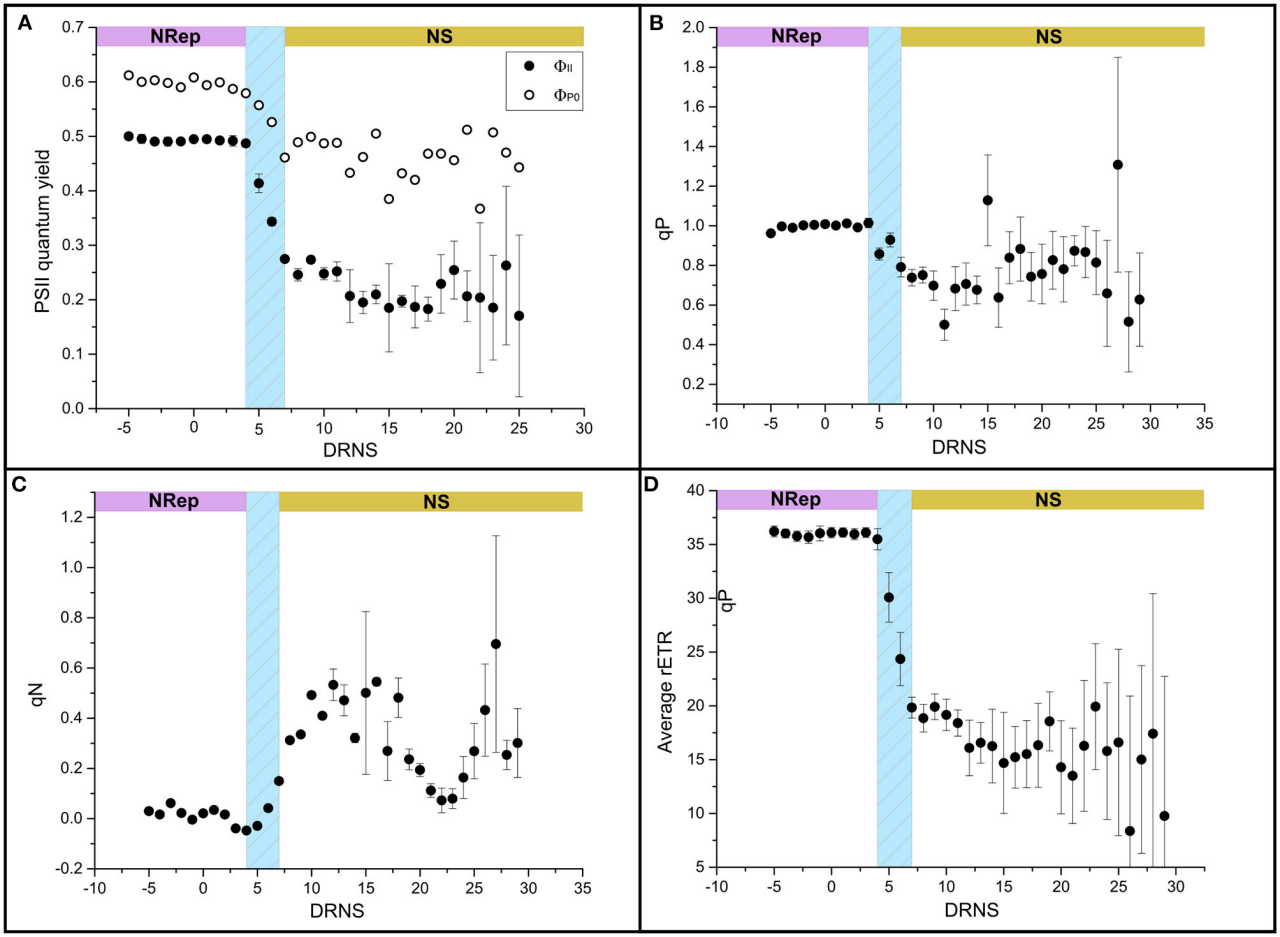
Impact of NS on the photosynthetic performance. **(A)** Light-adapted state (ΦII) (closed symbol) and dark-adapted state (ΦP0) (open symbol), **(B)** photochemical quenching (qN), **(C)** photochemical quenching (qP), and **(D)** relative electron transport rate (rETR).

### Effects of the Nitrogen Starvation on the Cellular Energy Content

Carbohydrates, lipids, and proteins are potential substrates for respiration and represent the three major macromolecule classes used to E_a_. The kinetic of the E_a_ shows two increasing phases separated by a transient pause (ANOVA 95%, *p* = 0.8) (Figure 1C). The first increase depends on the transition phase and consists in the accumulation of both carbohydrates and neutral lipids (Figure 1D). A pause occurred at the onset of the NS phase with no significant E_a_ increase (ANOVA 95%, *p* = 0.8; Figure 1C). E_a_ accumulation resumed with the only energy accumulation in the form of neutral lipids (Figure 1D).

Soluble proteins cell quotas decreased gradually from the onset of the transition phase, negatively contributing to the energy storage (Figure 1D). In the present experiment, the major source of carbon as building blocks for these high-energy-containing molecules should have been DIC. However, DIC increased rapidly during the transition phase and has continued to increase in NS with a reduced rate (Figure 1E), while C-int displayed a small increase (9.3%), not coherent with the high lipid accumulation (Figures 1C,D). These observations suggest that the metabolic reorientation relies on carbon reallocation mechanisms rather than carbon fixation. To confirm the hypothesis of reduction in carbon fixation and bring evidence on the carbon reallocation theory, the photosynthetic activity and respiration activities were assessed.

### Impact of the Nitrogen Starvation on the Photosynthetic and Respiratory Activities

In NRep, the cellular pigment quota remained stable and reduced to 37% of the maximum in NS conditions (Table 2). The reduction of the pigment cellular quota has direct effects on light energy harvesting and it can be a direct response to the changes in photosynthetic efficiency. Chlorophyll fluorescence was used as a proxy of photosynthetic efficiency (Roháček et al., 2008). Both the dark-adapted state (Φ_P0_) and light-adapted state (Φ_II_) decreased during the transition phase, reaching an average of 0.48 and 0.23 respectively (Figure 2A), indicating a reduced photochemical utilization of the light energy. The photochemical quenching (qP) that indicates the actual photochemical capacity of PSII in the light-adapted state decreased from 100% to 60–70%, suggesting a decrease in the capacity of reopening the PSII after excitation (Figure 2B). This generated a need to dissipate the excess of the absorbed energy as heat as revealed by the transient increase of the non-photochemical quenching (qN) from 5 to 15 DNRS (Figure 2C). A pattern similar to Φ_P0_ was observed for the relative electron transport rate (rETR) (Figure 2D).

The daily average dissolved oxygen rapidly decreased at the onset of the transition phase (Supplementary Figure 4A). The decrease in dissolved oxygen has a negative linear correlation with the increase in DIC (Supplementary Figure 4B), suggesting that the factor influencing the DIC affects also the dissolved oxygen. Since a negative linear correlation was observed between DIC and photosynthetic performance (Supplementary Figures 5A,B), the same correlation analysis was performed between the dissolved oxygen and the same two photosynthesis parameters showing a positive relation in both cases (Supplementary Figures 5C,D). A decrease in dissolved oxygen is thus due to the decrease in its production by the reduced photosynthetic activity and its consumption by the respiratory activity.

### Effect of a Small N Pulse on Cell Growth and Biochemical Composition

To get information on the capability of the cell to recover from NS, a nitrogen pulse was applied to the culture after 31 DRNS to reach a final nitrate concentration of 0.35 mM. Supplementary Figure 6 shows the fast response of the cells to the sudden increase in N-ext. Within 24 h, cells remobilized the energy stored in lipids to transiently synthesized proteins and pigments, resuming cell division after a lag phase of at least 3 days as well.

### Transcriptomic and Metabolomic Analyses

**Figure 3:**
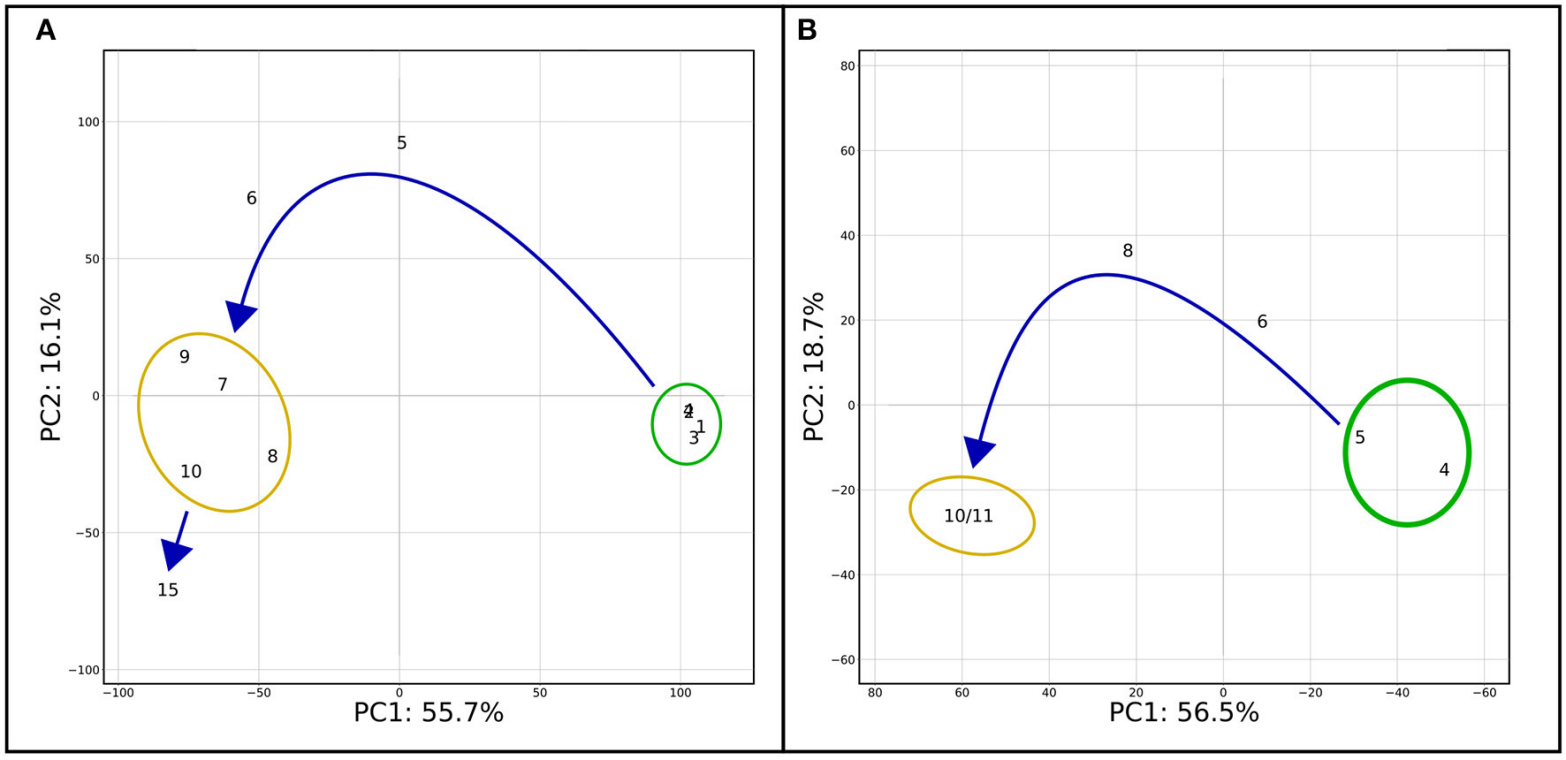
PCA on transcriptomic and metabolomic results. **(A)** PCA was performed on the significantly differentially expressed genes (|log2FC| ≥ 1). The first two components of the PCA explained 71.8% of the variance. **(B)** PCA performed on metabolites metabolite abundance compared to the control (before DRNS) during the time course of the experiment. Only metabolites which change is significant (|log2FC| ≥ 2) in at least one of the time points were considered for the analysis. As for Figure 1A, two major groups are delimited: NRep (green) and NS (yellow) with a transition phase in between. Differently from Figure 1A, day 15 has been considered as separate point and not together with the NS group.

**Figure 4:**
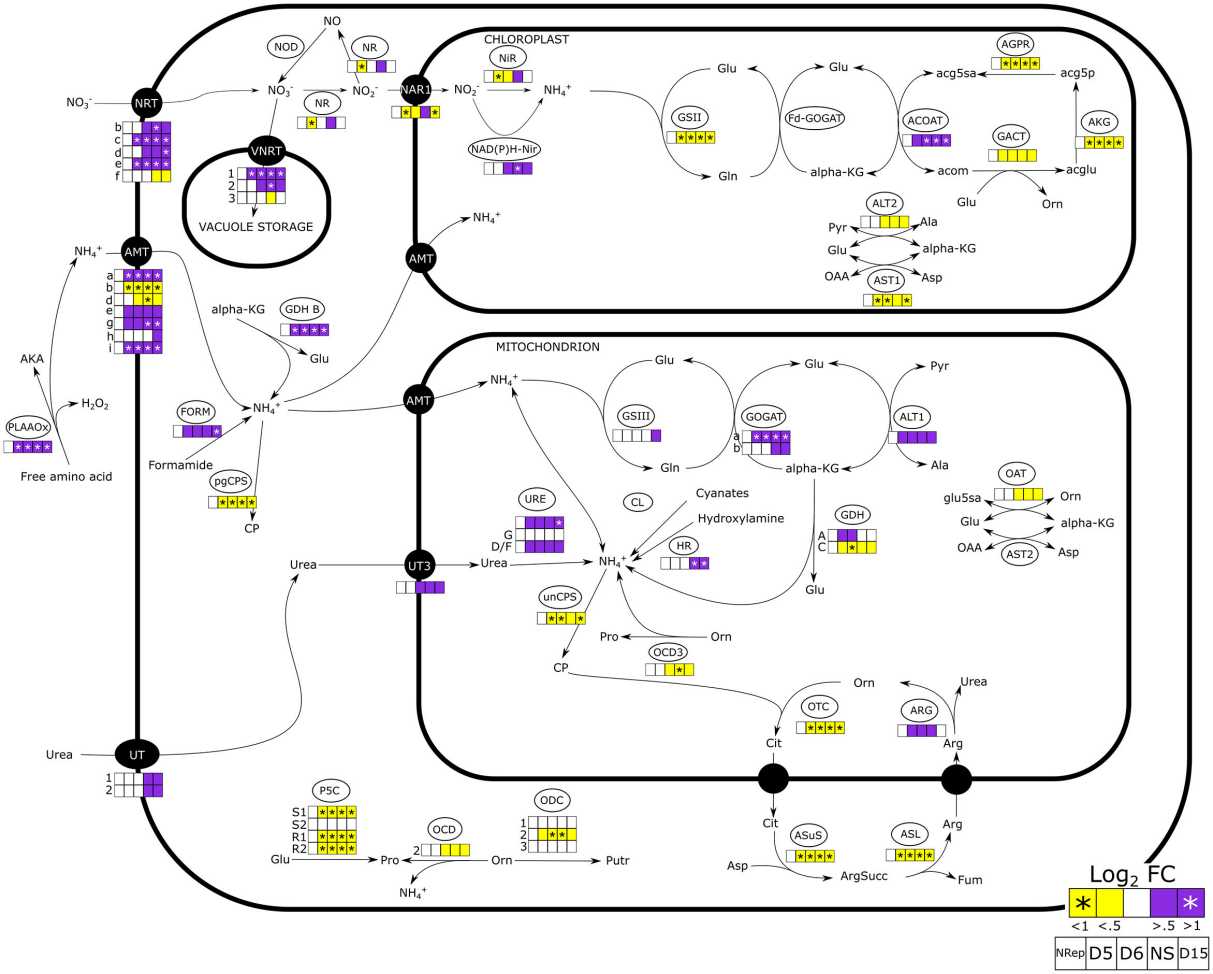
Nitrogen uptake and assimilation pathways in *Phaeodactylum tricornutum*. The heatmaps report the change in gene expression in the 5 different groups defined through the PCA analyses (Nrep, D5, D6, NS, and D15). Daily variations of gene expression are reported in Supplementary Figure 7. NRT, nitrate transporter; VNRT, vacuolar nitrate transporter; NR, nitrate reductase; NAR1, nitrite transporter; Nir, nitrite reductase; NAD(P)H-Nir, nitrite reductase NAD(P)H dependent; GSII/III, glutamate-ammonia ligase; GOGAT, glutamate synthase; ACOAT, acetylornithine aminotransferase; AGPR, N-acetyl-gamma-glutamyl-phosphate reductase; GACT, glutamate N-acetyltransferase; AKG, acetylglutamate kinase; ALT, L-alanine transaminase; AST, aspartate aminotransferase; PLAAOx, periplasmic L-amino acid oxidase; FORM, formamidase; GDH, glutamate dehydrogenase; CP, carbamoyl phosphate; pgCPS, carbamoyl phosphate synthetase; unCPS, carbamoyl phosphate synthase mitochondrial, UT, urea transporter; URE, urease; CL, cyanate lyase, HR, Hydroxylamine reductase; GDH, glutamate dehydrogenase; OCD, ornithine cyclodeaminase; ODC, ornithine decarboxylase; P5CS, pyrroline-5-carboxylate synthase; P5CR, pyrroline-5-carboxylate reductase; OTC, ornithine transcarbamylase; ARG, arginase; ASL, argininosuccinate lyase; ASuS, argininosuccinate hydratase; AKA, α-keto acid.

**Figure 5:**
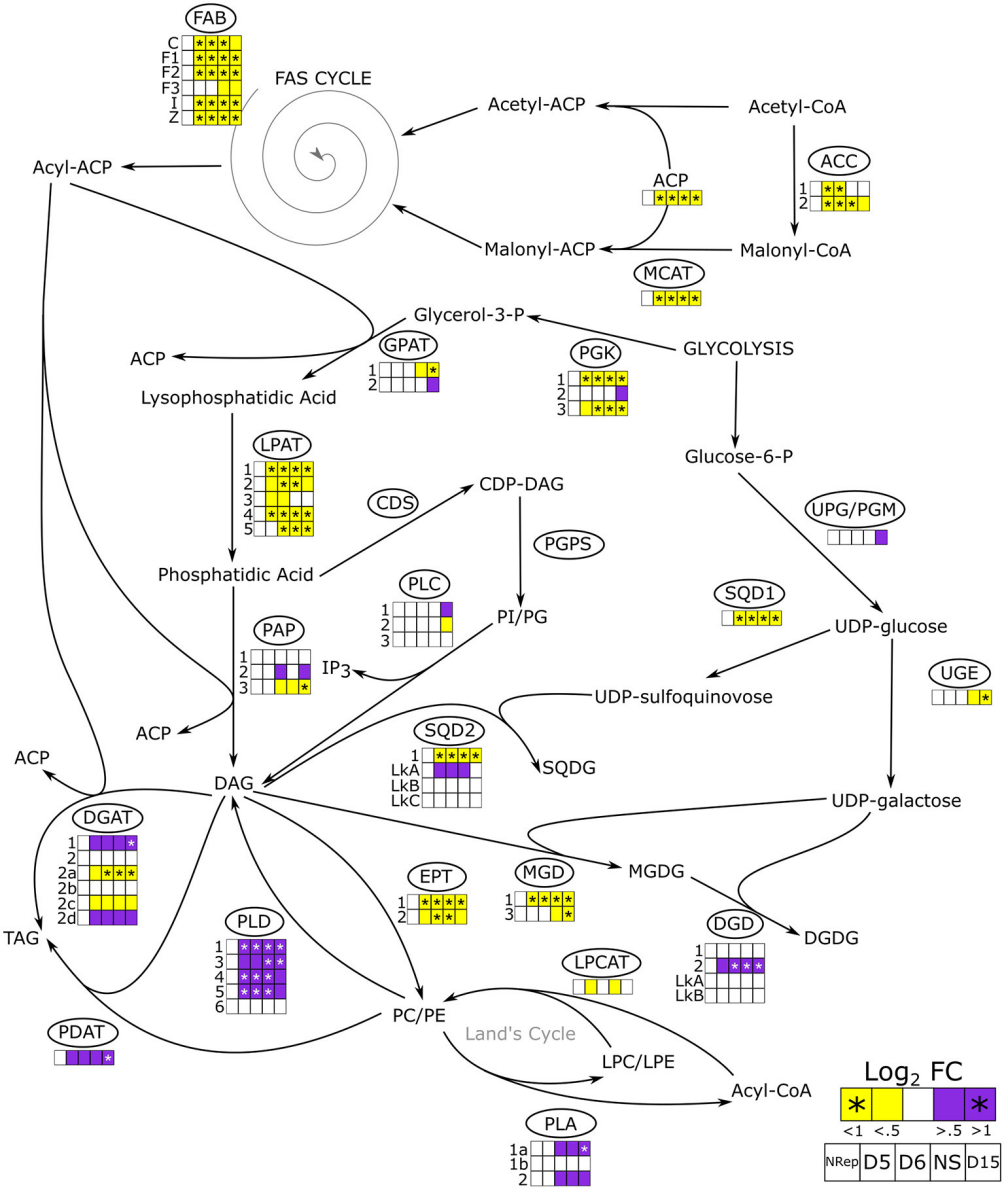
*De novo* and acetyl-CoA independent lipid biosynthetic pathways in *P. tricornutum*. The overall change in gene expression at each phase. PDC, pyruvate dehydrogenase complex; ACC, acetyl-CoA carboxylase; MCAT, malonyl-CoA:ACP transacylase; FAB, fatty acid biosynthesis; PGK, phosphoglycerate kinase; GPAT, glycerol-3-phosphate: acyl-ACP acyltransferase; LPAT, lysophosphatidate acyltransferase; PAP, phosphatidate phosphatase; CDS, phosphatidate cytidylyltransferase; PGPS, CDP-diacylglycerol–glycerol-3-phosphate 3-phosphatidyltransferase, PLC, phospholipase C; SQD1, UDP-sulfoquinovose synthase; SQD2, glycosyl transferase; UPG/PGM, UDP-glucose-pyrophosphorylase/phosphoglucomutase; UGE, UDP-galactose 4-epimerase; MGD, MGDG synthase; DGD, DGDG synthase; EPT, ethanolamine phosphotransferase; PLD, phospholipase D; PLA, phospholipase A; LPCAT, lysophosphatidylcholine acyltransferase; PDAT, phospholipid:diacylglycerol acyltransferase; DGAT, acyl CoA:diacylglycerol acyltransferase; ACP, acyl carrier protein. The heatmap is reported only when a change in gene expression is observed in at least one of the isoforms. Daily variations of gene expression are reported in Supplementary Figure 11.

**Figure 6:**
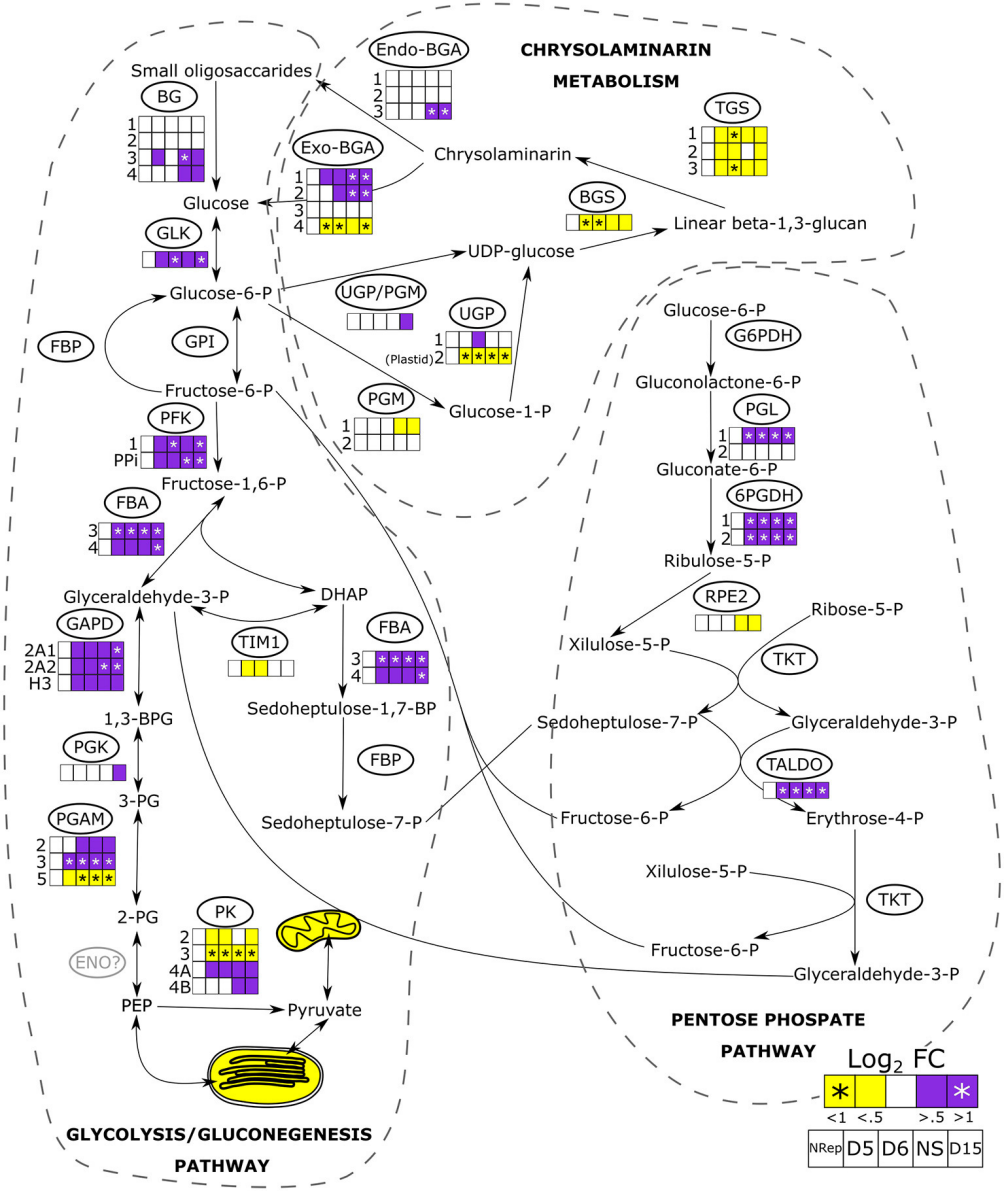
Transcriptional changes of the genes involved in glycolysis/gluconeogenesis, pentose phosphate pathways, the synthesis and degradation of chrysolaminarin. Heatmaps are reported when changes in gene expression were observed in at least one of the isoforms. Mitochondrial and plastidial transcriptional changes are reported as overall change, the detailed pathway and transcript abundance change are reported in Supplementary Figure 12. Full information on the gene expression change during the time-course of the experiment is reported in Supplementary Figure 13. BG, β-glucosidase, GLK, glucokinase; GPI, glucose-6-phosphate isomerase; FBP, fructose-1,6-bisphosphatase; PFK, phosphofructokinase; FBA, fructose bisphosphate aldolase; GAPD, glyceraldehyde-3-phosphate dehydrogenase; PGK, phosphoglycerate kinase; PGAM, phosphoglycerate mutase; ENO, enolase; PK, pyruvate kinase; TIM, triosephosphate isomerase; G6PDH, glucose 6-phosphate dehydrogenase; PGL, 6-phosphogluconolactonase, 6PGDH, phosphogluconate dehydrogenase; RPE2, ribulose-phosphate 3-epimerase; TKT, transketolase; TALDO, transaldolase; UPG/PGM, UDP-glucose-pyrophosphorylase/phosphoglucomutase; PGM, phosphoglucomutase; UGP, UTP–glucose-1-phosphate uridylyltransferase; BGS, 1,3-beta-glucan synthase; TGS, 1,6-β-transglycosylases; Exo-BGA, exo-β-glucanase; Endo-BGA, endo-β-glucanase.

On the mindset of investigating the effects and mechanisms underlying the NRep-NS transition, transcriptomic analyses were performed on 11 time points. To observe the changes induced by the reduction of nitrogen supply (RNS), the changes in gene transcript availability were expressed compared with the control NRep. Overall, 2,560 genes were differentially expressed in at least one DNRS (Supplementary Dataset 1). The PCA based on transcriptomic data (Figure 3A) exhibits a pattern very similar to the PCA based on the biochemical and physiological data (Figure 1A). However, they differ by the positioning of 15 DNRS that is hereby detached from the NS phase group, suggesting that important changes in gene expression occurred at this time point. For the sake of simplicity in the figure representation, five phases will be considered: (i) NRep, (ii-iii) 5 and 6 DRNS, (iv) NS, and (v) 15 DRNS. In the case of NRep and NS, the average transcript levels across the corresponding DNRS were calculated for each gene for heatmaps creation (Figures 4–6). To strengthen the information given by the transcriptomic analysis, metabolic profiling analysis was performed through LC-MS on samples corresponding to 4, 5, 6, 8, 10, and 11 DNRS, which all allowed the detection of 4,267 compounds (*p*-value ≤ 0.05) (Supplementary Dataset 2). Individual compound cell quota change kinetics showed that 2,867 compounds displayed a significant variation (|log2FC| ≥ 2) compared to controlled conditions (before DRNS) for at least one time point. A PCA analysis was performed taking into account these 2,867 compounds (Figure 3B), thus, resulting in a similar pattern as observed on a transcriptomic level. A little shift was observed corresponding to the translation of the transcript changes at the metabolic level, and a great change observed was also observed on days 10 and 11.

#### Nitrogen Uptake, Scavenging, and Assimilation

At the onset of the transition phase, the transcript availability of genes encoding for plasma membrane nitrate and ammonium transporters has increased. The upregulation of the urea transporters (UT) encoding genes was observed when cells entered the NS phase (Figure 4). Similarly, an increase in the transcript levels is also observed in two of the three genes encoding for the vacuolar nitrate transporters. On the contrary, the expression level of the gene encoding the plastidial nitrite transporter (NAR1), nitrate reductase (NR), and the plastidial nitrite reductase (NiR) displayed a peculiar expression pattern, with a decrease during the transition phase and overexpression in NS.

Genes involved in the mitochondria ammonia fixation were mostly upregulated from the onset of the transition phase. A strong upregulation of the gene encoding the cytoplasmic glutamate dehydrogenase (GDH-B: J13951) was observed as soon as the NS state was reached. An increase in transcript levels was observed for the mitochondrial glutamine oxoglutarate aminotransferase (GOGAT) but not for the plastidial GOGAT encoding gene (Figure 4).

External amino acids could serve as an alternative nitrogen source (Flynn and Butler, 1986). In this concern, the periplasmic L-amino acid oxidase (PLAAOx) catalyzes the cleavage of the ammonium group from free amino acids (Thiriet-Rupert et al., 2018). The transcript levels of the PLAAOx encoding gene greatly increased from the onset of the transition phase and remained upregulated during NS. However, a significant increase in PLAAOx transcripts starting from 4 DRNS suggested a potential role in cell-sensing of N level. Thiriet-Rupert et al. (2018) reported that the PLAAOx encoding gene is coexpressed with the gene encoding the coccolith scale associated protein (CSAP) during NS in *Tisochrisis lutea*, which was also observed in *P. tricornutum* (*R* = 0.9963, *R*^2^ = 0.9926).

An increase in transcript levels was observed for genes encoding proteins involved in nitrogen recycling such as deaminases, acetamide, and formamidase (Alipanah et al., 2015). The genes encoding for transporters of different nitrogen-containing molecules, such as polyamines and small polypeptides, were also upregulated in NS (Supplementary Dataset 1).

#### Amino Acid Metabolism

The total cell protein quota started to decrease after the RNS, reflecting both an increase in protein degradation and a decrease in protein synthesis. Upregulation of the genes encoding ubiquitin ligases, the enzymes that initiate the protein degradation and two 26S proteasome regulatory subunits (Subunit N3, EG02475, isoform 2 of the subunit 5, and EG0162) was observed in NS. Moreover, an increase in transcript levels encoding components of the ubiquitin-mediated protein degradation pathway was observed (Supplementary Dataset 1). Coherently, a decrease in transcript abundance for genes encoding aminoacyl-tRNA synthetases, ribosomal proteins, while translation and elongation factors were also observed. The overall transcript availability of genes involved in the biosynthesis of amino acids was reduced in NS conditions (Supplementary Dataset 1), with the sole exception of the serine and glycine, glutamine and glutamate biosynthetic pathway involved in photorespiration and central metabolism (Supplementary Figures 8, 9).

Branched-Chain amino acids (BCAA) have been reported to be involved in the accumulation of triacylglycerols (TAG) (Ge et al., 2014; Liang et al., 2019; Pan et al., 2020). Most of the genes involved in the BCAA degradation were upregulated (Supplementary Figure 10). In particular, the transcript abundance of the gene encoding the dihydrolipoyllysine-residue (2-methylpropanoyl) transferase (DHLTA, J54219), increased over 6 folds, remaining upregulated all throughout the transition and NS phases (D15 included) (Supplementary Figure 10). Transcript abundance of two genes encoding propionyl-CoA carboxylase carboxylases [PCC1 (J51245) and PCC2 (J45886)] started to be upregulated at the onset of the transition phase peaking during the NS. Furthermore, an increase in the transcript level was observed in the gene encoding the methylcrotonyl-CoA carboxylase (MCC2) (shikimate pathway) (Supplementary Figure 10). Pan et al. (2017) reported MCC2 as a key enzyme for which the silencing of the encoding gene (J19329) caused a decrease of carbon flux toward the TCA cycle, resulting in a decrease in TAG accumulation and nitrogen utilization. A change was also observed for the 3-hydroxyisobutyryl-CoA hydrolase (HIBCH) transcript abundance, which significantly increases in NS (Supplementary Figure 10).

Aside from proteins, other molecules such as polyamines require N for their synthesis. Polyamines are playing an essential regulatory function and are synthesized in all organisms including diatoms (Lin and Lin, 2019). The overall transcript abundance of the genes involved in the synthesis of the polyamines was decreased from the onset of the transition to the establishment of the NS phase (Supplementary Dataset 1).

#### Lipid Metabolism

Figure 5 presents the transcript abundance changes for the genes involved in lipid metabolism including *de novo* FA biosynthesis and the acetyl-CoA independent pathway.

##### *De novo* Biosynthesis

*De novo* FA biosynthesis depends directly on the plastid-residing acetyl-CoA pool, which is most likely the primary substrate for this pathway in diatoms (Zulu et al., 2018). *De novo* FA biosynthesis begins with the condensation of acetyl-CoA and malonyl-CoA, which are gradually added to the forming acyl molecule through the FA synthesis (FAS) cycle resulting in the acyl-CoA formation (Wang et al., 2018; Zulu et al., 2018). Then, the acyl groups are sequentially attached to the glycerol molecule, generated by the activity of the phosphoglycerol kinase (PGK) to form both storage and structural lipids (Figure 5). The whole genes encoding for components of the *de novo* FA biosynthesis were strongly downregulated (Figure 5). Moreover, the transcript abundance of most of the genes encoding enzymes, that involved in the acylglycerols formation, were reduced all along with the transition phases and the NS phase. In particular, all the five genes encoding lysophosphatidic acid acyltransferase (LPAT), responsible for the intermediate conversion of lysophosphatidic acid to phosphatidic acid, were significantly downregulated. The downregulation of the LPAT encoding gene under NS conditions has been observed by Alipanah et al. (2015), in opposition with what was observed previously by Valenzuela et al. (2012). The final conversion of DAG to TAG is carried out by the action of diacylglycerol *O*-acyltransferases (DGAT) encoded by six paralogous, four of which are differentially regulated. Two are slightly upregulated (DGAT1 and DGAT2D) while the other two are downregulated (DGAT2A and DGAT2C) in NS. The upregulation of DGAT1 and DGAT2D under NS starvation has been also observed by Alipanah et al. (2015). On the other hand, Remmers et al. (2018) reported no significant upregulation of the six DGAT encoding genes suggesting that the activity of these enzymes was not a limiting factor during steady-state growth under N-limitation conditions.

##### Acetyl-CoA Independent Biosynthesis

Besides the *de novo* FA biosynthetic pathway, a second pathway for FA synthesis is present in diatoms: the acetyl-CoA independent. The latter does not require the presence of an acetyl-CoA pool for the synthesis of acyl-CoA but recycles it from pre-existing polar lipids (Cagliari et al., 2011) (Figure 5). Membrane lipids including phosphatidylcholine (PC), phosphatidylethanolamine (PE), phosphatidylinositol (PI), and phosphatidylglycerol (PG) are hydrolyzed by phospholipases. Phospholipases C (PLC) and phospholipases D (PLD) are responsible for the release of DAG from PI/PG and PC/PE, respectively (Alipanah et al., 2018). While the negligible change in transcript abundance was observed for the genes encoding PLC, a strong upregulation was observed for genes encoding the PLD. Five out of 6 PLD encoding genes were strongly upregulated from the onset of the transition throughout NS (Figure 5). The released DAG can serve as a substrate for TAG formation through the activity of DGAT and PDAT. The latter then catalyzes the transacylation of an acyl group in position *sn-*2 of the PC to position *sn-*3 of the DAG forming TAG and LPC. The transcript abundance of the single PDAT gene in *P. tricornutum* started to increase at the onset of the transition phase, reaching a maximum in NS. Another acyl group recycling pathway is the Land's cycle (Lands, 1960; Zulu et al., 2018) (Figure 5), which involves the hydrolysis of PC and PE in Lyso-PC (LPC) and Lyso-PE (LPE), respectively, by the activity of the phospholipases A (PLAs). The second reaction that closes the cycle is catalyzed by the activity of the acyl-CoA:lysophosphatidylcholine (LPC) acyltransferase (LPCAT), which leads to the reformation of PC and PE. While one single LPCAT has been identified in *P. tricornutum* (Bowler et al., 2008), two PLA transcripts have been identified encoding for two different PLAs: PLA1, which cleaved an acyl group in position *sn*-1 from a PC releasing the acyl-CoA and LPC, whereas PLA2 cleaved the acyl group in position *sn*-2. Transcript abundance of genes encoding PLA1a and PLA2 significantly increased at the end of the transition phase (7 DRNS) and remained upregulated throughout the NS. In line with what was reported by Abida et al. (2015), a decrease in transcripts encoding MGDG synthase (MGD) and an increase in DGDG synthase (DGD) was observed, suggesting a reduction in the MGDG synthesis and its conversion to DGDG for further degradation. Decrease of membrane lipids, especially plastidial membrane lipids such as MGDG and DGDG, was also observed from a metabolomics point of view (Supplementary Dataset 2). Moreover, two putative DAG forms were shown to decrease in the NS phase and are in line with the hypothesis of the remodeling of lipids toward energy storage ones (Supplementary Dataset 2).

##### Lipid Storage

Due to their non-polar nature, neutral lipid accumulation requires proper storage in hydrophobic subcellular structures called lipid droplets (LD), which are characterized by specific LD proteins (LDP) (Goold et al., 2015; Lupette et al., 2019; Leyland et al., 2020). Two different LDP are present in *P. tricornutum*, StLDP (Stramenopiles LDP) and in PtLDP1 (*P. tricornutum* LDP1) (Wang et al., 2017; Lupette et al., 2019; Leyland et al., 2020). While PtLDP1 transcript abundance gradually decreased from the onset of the transition phase with a minimum at 15 DRNS, the StLDP encoding gene is overexpressed, mirroring the PtLDP1 expression pattern (Supplementary Dataset 1). Wang et al., (2017) reported that PtLDP1 upregulation resulted in an upregulation of DGAT2, GPAT, and FAS encoding genes, while in this experiment, an overall downregulation is observed for these genes (Figure 5). Moreover, a putative seipin homologous encoding gene (J47296) was mostly upregulated during the transition phase (Supplementary Dataset 1). The activity of seipins is important for LD biogenesis and maturation (Lu et al., 2017).

#### The Central Metabolism

In diatoms, the cytosolic glycolysis pathway is partially duplicated in the mitochondria and the chloroplast (Kroth et al., 2008). When the N-ext became minimum at 5 DRNS (Figure 1), genes encoding for components of both organelle pathways started to be downregulated (Figure 6). While most of the glycolytic enzymes catalyzed also a reverse reaction in the gluconeogenesis, the phosphofructokinase (PFK) and pyruvate kinase (PK) are unidirectional enzymes, and the overexpression of their relative genes suggests an increase in C flux toward the catabolic pathway, even though two of the four PK genes were downregulated (Figure 6). Kroth et al. (2008) reported the presence of a plastidic enolase (ENO) and two other non-functional mitochondrial ones, but no cytosolic ENO has been described so far.

Other pathways parallel to glycolysis exist: one of these being the pentose phosphate pathway (PPP) (Kroth et al., 2008) and the other is an alternative to PPP which involves the xylulose-5-phosphate/fructose-6-phosphate phosphoketolase (XPK) (Fabris et al., 2012). The gene encoding this enzyme that is involved in these two pathways was upregulated only during the transition phase (Supplementary Dataset 1). Pyruvate, the product of glycolysis and alternative pathways, can then enter the tricarboxylic acid cycle (TCA) *via* two ways (Supplementary Figure 13): (1) through the conversion to acetyl-CoA by the activity of the pyruvate dehydrogenase complex (PDH), or (2) through the conversion to oxaloacetate by the action of the pyruvate carboxylase (PYC). The overall genes involved in the TCA were upregulated in NS, with an overall expression peak at 8 DRNS (Supplementary Figure 13).

#### β-glucans Metabolism

The β-1,3-glucans biosynthetic pathway (Figure 6) starts with the formation of glucose-1-phosphate catalyzed by the phosphoglucomutase (PGM), which transfers the phosphate of the glucose-6-phosphate from position 6 to 1. The conversion of glucose-1-phosphate to uridine diphosphate glucose (UDP-glucose) is catalyzed by the cytoplasmic UDP-glucose pyrophosphorylase (UGP) (Kroth et al., 2008). Then, UDP-glucose serves as a substrate for the linear polymerization catalyzed by the activity of β-1,3-glucan synthase (BGS, single gene). This reaction generates a linear β-1,3-glucan that can be further processed to β-1,3/1,6-glucan polymer, which is called chrysolaminarin. The last reaction is catalyzed by β-1,6-transglycosylases (TGS, 3 genes). The chrysolaminarin breakdown to glucose molecules is carried on by endo- and exo-β-glucanases (endo-BGS, exo-BGS) (Figure 6). Small oligosaccharides are further hydrolyzed to glucose by the activity of β-glucosidases (BG), glucose feeding the central metabolism.

An overall downregulation of the genes involved in chrysolaminarin biosynthesis was observed in the NS phase (Figure 6), while a significant increase in transcript level was observed for genes encoding the enzymes catalyzing the breakdown of β-1,3-glucans, which increase during the transition phase. They remained elevated all along the NS phase (Figure 6).

#### Photosynthesis and Carbon Fixation

##### Light Harvesting

Light harvesting complex (LHC) proteins are functional components of photosystems having different functions including light harvesting, photosystem regulation, and photoprotection (Bassi and Caffarri, 2001; Scarsini et al., 2019). In NS, most of the LHC genes were downregulated with few exceptions including *LHCX4* and *LHCR10*. These two transcripts, together with the high-light inducible protein 1 (*HLIP1*) transcript, were immediately overrepresented at the onset of the transition phase (Figure 1). Four other genes were upregulated in these conditions: *LHCF6, LHCF9*, and *LHCF15* were transiently upregulated during NS, whereas *HLIP1b* was transiently upregulated at the onset of NS. An overall strong downregulation was observed for the nuclear-encoded PSII reaction center (*psb*) genes involved in photosynthesis and photosynthetic electron transport chain (Supplementary Dataset 1). The only observed exception was the gene encoding the iron-sulfur clusters of the cytochrome *b*_6_*/f* complex (PETC) that mediates the electron transfer between PSII and the PSI: it was upregulated during NS.

##### Photochemistry and Carbon Assimilation

The majority of the transcripts involved in photosynthesis were downregulated, in particular, a strong reduction was observed for the gene encoding the chloroplastic ferredoxin-NADP reductase (J23717) responsible for the photosynthetic production of NADPH, confirming the observations reported by Alipanah et al. (2015). In general, NS induced a strong reduction in transcript availability of most of the genes involved in the Calvin-Benson-Bassham cycle (Supplementary Dataset 1), while an overall increase in transcript abundance was observed for genes involved in the central PEP-pyruvate hub and for genes involved in the biochemical C4 cycle. High expression levels in NS were observed for the genes encoding the phospho*enol*pyruvate carboxylase 2 (PEPCase2), as well as its antagonist, the phospho*enol*pyruvate carboxykinase (PEPCK).

An increase in the transcript levels of genes encoding the malic enzyme (ME) and the 2-oxoglutarate: malate exchanger (EG02645) was observed in NS with two major expression peaks at 8 and 15 DRNS. While the malate exchanger encoded gene was upregulated, an overall downregulation was observed for the 4 plastidial triose phosphate transporters encoding genes (TPT1, TPT2, and TPT4a/b; Moog et al., 2015).

Most of the carbonic anhydrase (CA) genes were downregulated especially in NS with the sole exception of the CA1, which gene expression doubled at the onset of the NS phase (Supplementary Dataset 1).

##### Photorespiration

Huang et al. (2015) observed an increase in serine and glycine *de novo* biosynthesis *via* photorespiration, highlighting the potential importance of photorespiration in N redistribution in NS conditions. Our transcriptomic analysis showed an increase in several gene encoding for components of the photorespiration pathway. In fact, an increase in the transcript levels of genes encoding for serine/alanine:glyoxylate aminotransferase (J49601 and J43874) and the gene encoding for the glycine decarboxylase (EG02292) was observed (Supplementary Dataset 1). These two enzymes respectively converted the toxic glyoxylate in glycine than in serine, releasing one molecule of CO_2_ and one molecule of ammonia (Kroth et al., 2008; Kunze and Hartig, 2013) (Supplementary Figure 8).

##### Photosynthetic and Photoprotective Pigment Synthesis

As expected, in NS, the *P. tricornutum* culture color faded from a pronounced brown-green to a very light yellow recalling the chlorotic phenotype observed in other photosynthetic organisms (Allen and Smith, 1969). The transcript abundance of most of the genes involved in the chlorophyll *a* biosynthetic pathway was decreased in this condition (Supplementary Dataset 1). The exception was observed for CHLC_2 and POR4, which were upregulated in NS. Simultaneously, genes encoding for the carotenoid biosynthesis were downregulated in NS. Moreover, lowering the N availability triggered a reduction of the transcripts levels corresponding to two main key enzymes of the xanthophyll cycle, violaxanthin de/epoxidase, and zeaxanthin epoxidase (Scarsini et al., 2019).

Moreover, metabolite elucidation showed that 6 putative compounds belonging to the carotenoid biosynthetic pathway decreased in abundance during the time course of the experiment and, being in line with what was observed on a transcriptomic level (Supplementary Dataset 1).

## Discussion

The described analyses were performed in highly controlled growing conditions, allowing to observe the response of *P. tricornutum* to the sole NS while avoiding other limitations. The highly controlled turbidostat mode cultivation allows maintaining specific culture conditions for a long period free from the biases of the batch and fed-batch mods (Mcgeachy et al., 2019), while the addition of fresh medium is controlled by cell division rate. In fact, the latter is linearly proportional to nutrient availability and *vice versa* (Yaakob et al., 2021). In the absence of the desired modifications, such as the removal of nitrogen from the supplied medium, cells are continuously submitted to non-limiting nutrient availability. If N is removed from the supplied medium, it will be the only growth-limiting nutrient. Other nutrients, such as phosphorous, remained stable as they are uptaken by the cells with a proportional rate to the culture growth (Yaakob et al., 2021). In general, under turbidostat, each cell is submitted not only to constant nutrients, except for the desired removal, but also to light supply. By keeping the cell concentration sufficiently low, the self-shading effect will be minimized (Carvalho et al., 2011). This scenario cannot be reproduced in a batch culture where growing conditions continuously evolve. Not only does the nutrient availability per cell gradually decrease, but light distribution is affected by the increase in cell concentration as well. Continuous cultivation methods such as chemostat and turbidostat are important for experiment reproducibility, not only on a biochemical and physiological, but as well in a molecular level (Regenberg et al., 2006; Daran-Lapujade et al., 2009).

### Intracellular Nitrogen Availability Guides the Passage Between Two Equilibria, NRep and NS

In nature, microalgae grow in very variable conditions and they may be submitted to reduced N availability that may eventually evolve into N starvation. As described above, the only modulated parameter is the N availability in the form of nitrate while all the other parameters are kept constant. The multidisciplinary approach allowed us to have a comprehensive view of the transition from NRep to NS. The broad picture of the metabolic reorientation, given by the PCA of transcriptomics, metabolomic and biochemical-physiological responses (Figures 1, 3), was crucial to highlight that the DRNS induces a shift from an equilibrium to another. As expected, an equilibrium is observed in NRep conditions since cells are not subjected to limitation. Interestingly, a second equilibrium is observed when NS is reached. Cell metabolism has been reoriented and no qualitative changes were observed. Independently from the timepoint, cells in the NS phase possess the same metabolic instruction set. The observed changes during the NS phase are, thus, only quantitative (*e.g.*, increased neutral lipid content), while qualitative changes (*i.e*., metabolic reorientation) have occurred during the transition phases.

The N-int appears to be the driving force that guides the shift between NRep and NS. In fact, cells remained in NRep phase until the minimum N-ext was reached and cells were forced to utilize the N-int. Division rate and N-int are closely interconnected (Figure 1B). In fact, the pool of N-int is split between the two daughter cells during cell division and the lower N-int will affect the cell division rate (Alipanah et al., 2015). An equilibrium is reached when the actual N availability is enough to maintain a minimum level of cell division rate. Even if the parameter guiding the NRep to NS transition is N-int, cells are not unaware of the changing environmental conditions before the trigger point. Indeed, 69 genes were differentially regulated at 4 DRNS including PLAAOx and CSAP encoding genes and one transcription factor of the heat shock family (HSF) (Supplementary Dataset 1).

### Decreasing Nitrate Availability Triggers Nitrate Uptake and Storage Rather Than a Direct Utilization

The time shift of the decay of N-int compared with N-ext (Figure 1) is due to the presence of an intracellular N storage pool (Shoman, 2015). Aside from organic N, present in proteins and other N-containing molecules, nitrate can be directly stored in the cells. Mccarthy et al. (2017) reported that cells possess a remarkable ability to rapidly transport, assimilate, and also, presumably, store ${\text{NO}}_{3}^{-}$ in vacuoles. The transient decrease of NR encoding transcript during the transition phase in contrast with increased transcript levels of genes encoding ${\text{NO}}_{3}^{-}$ transporters of both plasmalemma and tonoplast, confirms the hypothesis of the potential storage of ${\text{NO}}_{3}^{-}$ in the vacuoles (Figure 4). A temporary decrease in NR has been also observed by Longworth et al. (2016) within 72 h of NS.

Nitrate was the only N source in the medium, however, the effort of the cell to scavenge N from other N-containing molecules, such as free amino acids, is suggested by the upregulation of the gene encoding the PLAAOx. This enzyme oxidizes the aminoacidic groups to release ammonium which can be readily exploited by the cell as a source of N. The early overexpression of the PLAAOx and CSAP encoding genes (Figure 4), which has also been observed in *T. lutea* (Garnier et al., 2016), suggests a potential involvement of PLAAOx and CSAP in N sensing. Moreover, an alternative source of ammonium can be photorespiration which has already been observed as taking place in N-limited conditions (Huang et al., 2015). The increase in transcripts encoding, not only mitochondrial GOGAT but also serine/alanine:glyoxylate aminotransferase and glycine decarboxylase, leads to the conclusion that in NS, a cellular effort in ammonia assimilation is observed following what was introduced by Remmers et al. (2018). Amidase activity is also contributing to ammonia scavenging, facing N deprivation (Alipanah et al., 2015).

### Chloroplast Stand-by Mode as a Long-Term Cell Survival Mechanism

The passage to NS induces exposes cells to an energy imbalance between light availability and demand from cellular metabolism and growth (Remmers et al., 2018). The gradual development of the chlorotic phenotype is consistent with the decrease in transcript abundance of the majority of genes encoding LHCs, which is also reflected in the optimization of antenna size. On the other hand, the increased LHCX4 and LHCR10 transcript abundance suggests the need to increase the dissipation of excess absorbed energy. These two proteins belong to distinct families of LHCs, reported playing important roles in photoprotection (Lepetit et al., 2012; Collier Valle et al., 2014; Taddei et al., 2016; Buck et al., 2019). Both LHCX4 and LHCR10 encoding genes have been reported to be upregulated under nitrogen deprivation (Yang et al., 2013; Alipanah et al., 2015). While LHCR10 has been shown to be involved in photoprotection in response to the low light-to-high light transition of *P. tricornutum* (Collier Valle et al., 2014), LHCX4 does not appear to be involved in the fluorescence quenching photoprotection (Buck et al., 2019, 2021). Buck et al. (2021) suggested that one gene alone cannot contain all required regulatory *cis*-elements needed to respond to the multitude of environmental triggers and, thus, LHCX4 protein may play an integrative role between N starvation and photoprotection. Hence, the optimization of antenna size resulted in a decrease of the energy flow through the photosynthetic reaction centers, limiting photodamages and NADPH and ATP productions, the two key cofactors of the Calvin-Benson-Bassham cycle. In NS conditions, ribulose cannot be transformed into 3-phosphoglycerate but could enter the photorespiratory cycle (Sharkey et al., 1988; Bailleul et al., 2015). This is demonstrated by the increase of DIC (also observed by Valenzuela et al., 2012), the lack of increase in cell carbon quota (Figure 1C), the changes in expression of genes encoding Calvin-Benson-Bassham, and photorespiration components (Supplementary Dataset 1). This interpretation is further supported by the upregulation of the genes coding the enzymes involved in the photorespiratory pathway such as the alanine:glyoxylate aminotransferase (J49601, Supplementary Dataset 1).

A reduction of the plastidial activities follows a reduction of its energy content. Despite being exposed to low nitrogen availability for longer than 25 days, *P. tricornutum* was capable of a fast (within 24 hours) recovery of cell activities when a N source was reintroduced (Supplementary Figure 6). This fast recovery is incompatible with a complete shutdown and degradation of the chloroplast. These results confirmed previous observations made by Valenzuela et al. (2012). In short, NS turns chloroplast metabolism into a stand-by mode and, hence, the cell metabolism. In this scenario, the energy available in the chloroplast is not enough to keep it functioning and the mechanisms of organelle crosstalk need to be established as demonstrated by Bailleul et al. (2015). Indeed, the increase in the transcription levels for the gene encoding components of the malate valve (2-oxoglutarate:malate translocator and malic enzyme) suggests a connection between the different cellular pyruvate hubs and the energy transfer from the TCA to the plastid. Interestingly, the activation of the pyruvate hub (PEPCK, PEPC1, and PYC1) has been described as a default answer to the energy unbalance (Heydarizadeh et al., 2019), allowing the chloroplast stand-by mode as described in this work. This stand-by mode is reminiscent of the quiescent state described in *Chlamydomonas reinhardtii* (Tsai et al., 2018) and *Nannochloropsis oceanica* (Zienkiewicz et al., 2020). Regardless of their denomination, quiescent state, or standby mode, these mechanisms seemed highly conserved among microalgae and are crucial for cell survival in unfavorable conditions, such as those that may occur in nature. Deciphering these mechanisms may contribute to speeding up the development of microalgae as cell factories for the production of metabolites.

### The Intricated Response of the Central Carbon Metabolism to Nitrogen Starvation

Central carbon metabolism plays an important role in carbon partitioning mainly toward the synthesis of the major organic macromolecule classes (*i.e*., carbohydrates, proteins, and lipids), and plays a crucial role in response to environmental changes (Launay et al., 2020).

Different from TAG, the carbohydrate cellular quota is higher under NS but no continuous increase is observed (Figure 1D). In fact, Caballero et al. (2016) observed that *P. tricornutum* accumulates only 6% of cellular carbon in the form of chrysolaminarin in NS. In general, microalgae tend to break down storage carbohydrates in order to redirect carbon toward lipid biosynthesis (Hockin et al., 2012; Yang et al., 2013). Physiology and transcriptomic analyses showed a decrease in photosynthetic activity and carbon fixation through the Calvin-Benson-Bassham cycle, implying a decrease in *de novo* glucose generation and, thus, a reduced substrate for storage carbohydrates. The increase in carbohydrate cell quota, already reported in the literature (Caballero et al., 2016; Remmers et al., 2018), may suggest an increase in energy storage in the form of chrysolaminarin. However, according to the transcriptomic analysis, a decrease in chrysolaminarin biosynthesis and an increase in its breakdown could have occurred (Figure 6). The observed carbohydrate quota increase could be the effect of the active central metabolism suggested by the increase in transcript abundance of cytosolic glycolysis and TCA. The observed increase in carbohydrate quota is not continuous and occurred mostly during the transition phase. Thus, this increase is not due to an effort in carbohydrates storage but a carbon backbones remobilization and a central metabolism overactivity. The increase in transcript levels encoding two enzymes involved in gluconeogenesis, PEP carboxykinase (PEPCK) and pyruvate carboxylase 1 (PYC1), could suggest the neosynthesis of simple sugar and could explain the increase in carbohydrate cell quota. This hypothesis is corroborated by the observation of Matthijs et al. (2017) as an increase in maltotriose, maltose, and glucose in nitrogen-depleted *P. tricornutum* cells.

Remmers et al. (2018) stated that the central carbon metabolism response to NS is an intricate regulation of three oxidative pathways and gluconeogenesis. An increase in the transcript levels of the gene encoding components cytosolic classic glycolytic pathway is observed, suggesting an increase in glycolytic activity and confirming what was observed by Remmers et al. (2018) on both the transcriptomic and proteomic levels. Organelle counterparts are, on the other hand, downregulated. The increase in transcripts levels encoding components of the PPP, which produce NADPH (Kroth et al., 2008), has been observed. This pathway, together with chrysolaminarin degradation and glycolysis, suggested an increased effort of N starved cells to provide carbon fluxes through the TCA and balanced the reduced NADPH production from photosynthesis, as reported previously by Alipanah et al. (2015). The upregulation of genes encoding components of the TCA cycle also suggests an overactivity of answering the need of cells to compensate for the energy loss due to the reduced photosynthetic activity. Furthermore, TCA serves as a recycling of the carbon backbones derived from proteins and amino acids (Hockin et al., 2012). These catabolic activities produce oxaloacetate which can be converted in PEP by the activity of PEPCK or enter the malate shunt, contributing to the subcellular compartment energy balance (Selinski and Scheibe, 2019). Flügge et al. (2011) highlighted the crucial role of the PEP supply to plastids and its restriction, which may be eventually fatal for the plastid performance. Both the accumulation of TAG and carbohydrates, as well as protein degradation, are energy-requiring activities (Peth et al., 2013; Subramanian et al., 2013). Accelerating the central carbon metabolism allows the cells to both generate energy molecules and recycle carbon backbones during NS.

### Lipids Accumulation During NR-to-NS Involves Intracellular Carbon Remobilization Rather Than CO_2_ Fixation

The gradual accumulation of lipids and carbohydrates starts at the onset of the transition phase (Figure 1D). To explain this result, two alternative and non-exclusive hypotheses can be proposed: either the carbon required for the production of these molecules comes from the photosynthetic carbon fixation or/and from internal carbon reallocation. The data presented in this manuscript are in favor of the second hypothesis because all the genes involved in the FAS cycle are drastically downregulated, which would result in a strong reduction of the carbon flux through the *de novo* lipid biosynthesis pathway. The reduction in *de novo* FA biosynthesis has been previously observed in literature which supports the hypothesis of a lipid reallocation rather than biosynthesis (Valenzuela et al., 2012; Alipanah et al., 2015; Remmers et al., 2018). This reduction is attested by the strong increase in DIC, together with a decrease in photosynthesis (Φ_II_ and qP). Further proof of the reallocation of the carbon, rather than a fixation, is also explained by the very small percentage increase in cell carbon quota (Figure 1C), which does not fit with a *de novo* lipid biosynthesis deriving from carbon fixation. Moreover, the *de novo* fatty acid biosynthesis requires the acyl carrier protein (ACP), which expression is downregulated. Thus, in the absence of active carbon fixation, cells have no other choice but to convert pre-existing membrane lipids such as PC and PE. The choline and ethanolamine groups are removed by the action of the PLD, of which the majority of genes are upregulated in NS. The upregulation of genes encoding for the DGAT fits with an increase in TAG synthesis utilizing acyl groups, mostly generated by the breakdown of the phospholipids by the activity of the PLA, which encoding genes are upregulated. On the same frame, PDAT enzymes directly transfer an FA from the phospholipids to the DAG, while DGAT attaches a free FA group to DAG molecules. The same happens to the MGDG and DGDG, which are also a source of DAG and FA. Despite no gene encoding galactolipases have been so far identified in *P. tricornutum*, both transcriptomic and metabolomic data indicate an increase in galactolipid catabolism, thus, releasing fatty acids for TAG synthesis through lipid recycling.

## Conclusions

The interdisciplinary approach used to study the kinetics of the response of *P. tricornutum* to a gradual reduction in N availability allowed us to observe that cells shifted from one metabolic status, in which energy is mainly exploited for cell division, to another one, in which cells division rate is reduced in favor of energy storage. The reduction in N availability forced the cells to exploit the intracellular N pool and, thus, induced a dismantling of non-essential N-containing cellular components. Division rate is reduced in response to low N and storage molecules, mainly neutral lipids, are accumulated. Plastids are the subcellular components mainly affected by NS and their activity is reduced to a “standby mode” with a lower carbon fixation level. The response resided in the intricated modulation of the central carbon metabolisms which is the flywheel of the cell, allowing to maintain a basal energy status under these sub-optimal conditions. In fact, even after a long N limitation, cells are capable of fast remobilization of the stored energy to recover their function. *P. tricornutum* cells responded to NS, and generally to environmental stresses, with a mechanism similar to quiescence, in which energy is stored and non-essential activities are drastically reduced.

## Data Availability Statement

The transcriptomic data for this study have been deposited in the European Nucleotide Archive (ENA) at EMBL-EBI under accession number PRJEB47181 (https://www.ebi.ac.uk/ena/browser/view/PRJEB47181). The metabolic profiles are publicly available on the online infrastructure Ifremer Sextant catalogue of referential data from marine environments (https://doi.org/10.12770/38e00808-ffca-4266-943f-b691d3ca0bec). Data processing and statistical analysis of the metabolic profiles can be found at SANOE repository (https://doi.org/10.17882/84209). The data supporting the findings of this study are available from the corresponding author, Benoît Schoefs, upon request.

## Author Contributions

BS, JM, and MS: conceptualization. FM, JM, MS, and ST-R: data curation. BS, FM, JM, MS, and ST-R: formal analysis. BS, FM, HH, and JM: funding acquisition. BS, BV, JM, and MS: investigation. BS, BV, FM, JM, and MS: methodology. BS and JM: project administration. MS: software. BS, HH, and JM: supervision. BS, FM, JM, and MS: writing. All authors contributed to the article and approved the submitted version.

## Conflict of Interest

The authors declare that the research was conducted in the absence of any commercial or financial relationships that could be construed as a potential conflict of interest.

## Publisher's Note

All claims expressed in this article are solely those of the authors and do not necessarily represent those of their affiliated organizations, or those of the publisher, the editors and the reviewers. Any product that may be evaluated in this article, or claim that may be made by its manufacturer, is not guaranteed or endorsed by the publisher.
