# Supplementary material for: The Transition Toward Nitrogen Deprivation in Diatoms Requires Chloroplast Stand-By and Deep Metabolic Reshuffling
Source: Front Plant Sci. 2022 Jan 18;12:760516. doi: 10.3389/fpls.2021.760516 (PMC8811913; doi:10.3389/fpls.2021.760516)
Supplement: Supplementary file 1 [file Data_Sheet_1.docx]

# **Transition toward nitrogen deprivation in the diatom *Phaeodactylum tricornutum* highlights chloroplast stand-by and deep metabolic reshuffling**

Scarsini M.^1^, Thiriet-Rupert S.^1,2^, Veidl B.^1^, Mondeguer F.^3^, Hu H.^4^, Marchand J.^1^ and Schoefs B.^1^

**Supplementary Data**

Information S1

Figures S1-S21

Table S1-S3

### **Supplementary Information S1**

**Detailed information on metabolomic analyses -** LCHRMS/MS analyses were performed with an Agilent 1290 Infinity II LC system coupled to a high-resolution time-of-flight mass spectrometer (Q-Tof 6550 iFunnel, Agilent technologies, CA, USA) equipped with a Dual Jet Stream® electrospray ionization (ESI) source (positive mode). The operation parameters were the following: capillary voltage at 3900 V, fragmentor voltage at 150 V and skimmer at 60 V. The sheath gas was at 350 °C (12 mL min^−1^), the drying gas at 175 °C (5 mL min^−1^) and the nebulizer at 43 psi. Nitrogen was used as the collision gas. Mass spectra were acquired in full scan analysis over an m/z range of 50–1700 using the extended dynamic range and storage in centroid mode. The instrument resolution was 15,000 FWHM (Full Width at Half Maximum) at m/z 118.0862 and 30,000 FWHM at m/z 922.0098. To ensure the desired mass resolution, continuous internal calibration (Tuning Mix) was performed during analyses by using the signals at m/z 121.0509 (protonated purine) and m/z 922.0098 [protonated hexakis(1H,1H,3H–tetrafluoropropoxy) phosphazine (HP-921)] in the positive ion mode. For all samples analyzed, three replicates of each analytical sample were injected in the same batch and the order of sample injection was randomized to avoid sample bias.

Two quality controls were performed: (1) measured data from a certified reference materials of seven equimolar concentrations phycotoxins (Zendong et al., 2017) including okadaic acid (OA), dinophysistoxin-1 (DTX1), yessotoxin (YTX), azaspiracid-1 (AZA1), 13-desmethyl spirolide C (desMeC), gymnodimine (GYM), pectenotoxin-2 (PTX2) purchased from National Research Council Canada; (2) equal volumes of each sample were pooled together each sample and periodically analyzed throughout the samples analytical run. This will ensure that none or minimal metabolic information was lost.

Without additional preparation, aliquots (5 μL) of each sample were separated on a column “Acquity UPLC HSST3” (Waters) (2.1x150 mm, 1.7 μm), and the products were eluted at a flow rate of 200 μL min^−1^ using a gradient composed of solvents A (water-formic acid 100:0.001 v/v) and B (acetonitrile-formic acid 100:0.001 v/v), according to the following procedure: 0–3 min, 100% A; 3–8 min 0%–50% B; 8–13 min 50% B; 13–20 min 50–95% B;20–30 min, 95% B, 30–31 min 95–0% B, 31–36 min 100% B. In this study, several pre-processing steps (feature finding, alignment, filtration and normalization) were used to reduce the large number of data to an operational size and to obtain an adequate set of compounds before applying multivariate analysis.

Before process all the data obtained by QTOF-MS in full scan MS mode, acquired data was pre‐processed by the Agilent metabolomics software platform using Profinder (version B.10.0). A specific methodology was set to be as representative as possible of the total number of ions in samples. The parameters for pre‐processing were established based on the batch recursive feature extraction (RFE) for small molecules option. RFE included a molecular feature extraction (MFE) and then used mass and retention time (Rt) from the results to perform a targeted feature extraction referred to as Find by Ion (Step 8 of 8). The RFE was programmed based on: (1) extraction parameters for in input data: Rt (0.000–30.500 min), peak filter with height ≥ 300 counts, ion species (positive ions +H and +Na). (2) alignment parameters: Rt tolerance (0.10 min); mass tolerance (±10 ppm + 2.00 mDa). (3) MFE filters: absolute height (≥3000 counts), score (≥0.00%); (3) extraction ion chromatogram (EIC) tolerance (symmetric ppm ± 15 and ±0.5 min); (6) EIC peak integration and filtering (Agile2: Agilent integrate algorithm), FbI filters: absolute height (≥2000 counts); (8) FbI post processing filters (absolute height [≥1000 counts], score Tgt [≥50.00%]). A compound must satisfy the checked FbI in at least 2 files, in at least one sample group. Thus, out of a total of 15,073 ions the different sorting allowed to obtain a set of 6311 ions. This entity filtering allowed creating a higher quality data set so that the subsequent multivariate analysis could be more meaningful.

After pre‐processing data, data was exported to Mass profiler professional (MPP version 15.0, Agilent Technologies, Santa Clara, CA, USA) for processing and analysis. The parameters of Mass Profiler Professional were set as follows: Experiment type: Combined (Identified + Unidentified); Organism: None; Workflow type: Data Import Wizard. Total data set: 6263 - II Experiment - All Entities (Created from Significance Testing and Fold Change workflow step):

- 5427-Filtered on Flags (Entities retained in which at least 1 out of 27 sample has Present [P] or Marginal [M] Flag)

- 5427-Filtered by frequency (where at least 100.0 percent of samples in any 1 out of 6 conditions has flag [P])

- 4418-Oneway ANOVA, p (P-value cut-off ) <0.05

- 4267-Fold change >= 2.0 – (Pairing option: All conditions against control)

### **Supplementary Figure S1**


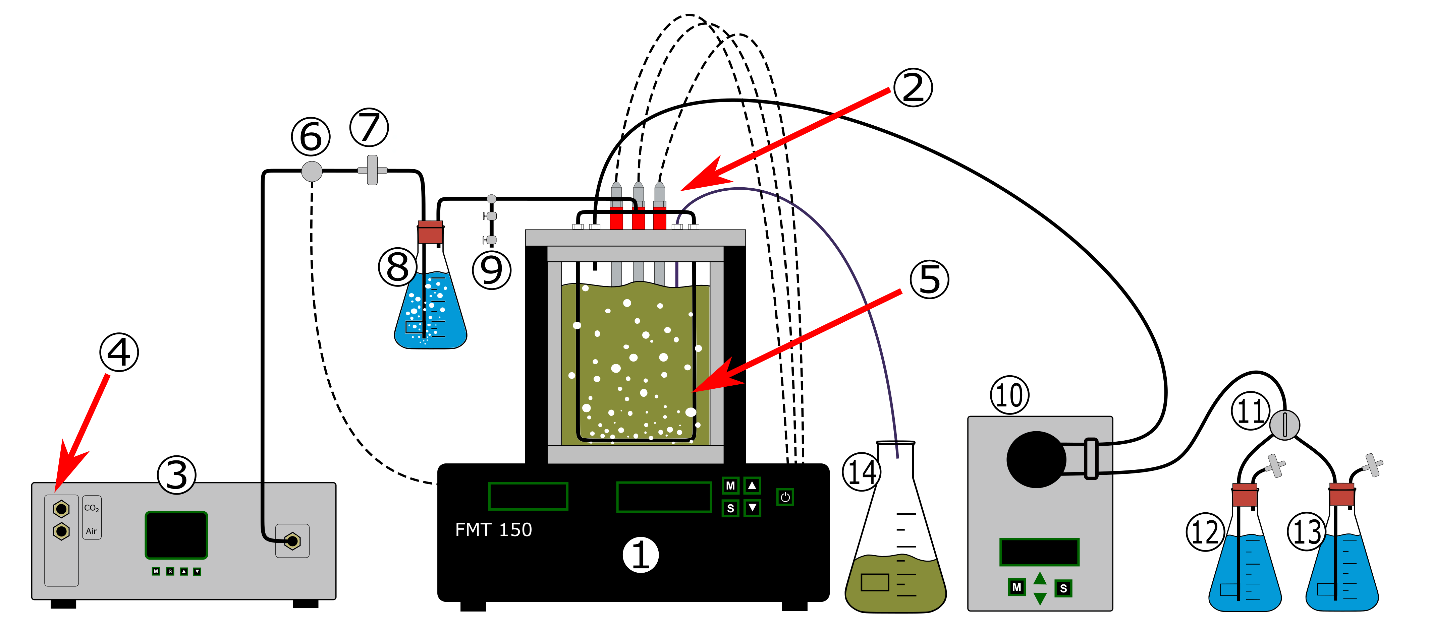


**Schematics of the cultivation setup.** (1) FMT 150 PBR central unit: it is composed of the cultivation chamber with all the electronic circuits for the PBR-computer interface. It also contains the led panel, the bubbling system, temperature control unit, the integrated densitometer and fluorometer for chlorophyll fluorescence analysis. In the upper part of the central unit, three sensors are visible (2) and are the dedicated sensors for temperature/pH, oxygen and DIC monitoring. On the left of the PBR is located the gas managing system (3). Gas mixing system GMS 150 used to precisely prepare the air/CO_2_ mixture before injection in the PBR bubbling system through the bubbling loop (5). Air and CO_2_ are supplied on the mixing system from bottles through the dedicated inlets (4). A solenoid valve (6) is electronically controlled by the system in order to interrupt the bubbling when requested (e.g. optical density reading). An air filter (7) is added to avoid potential impurities of the air/CO_2_ mixture to reach downstream compartments. A bottle containing ultrapure sterile water where the air/CO_2_ mixture to humidify it prior to enter the cultivation vessel (8). A sampling catheter (9). On the right side of the PBR is located the medium managing system. A peristaltic pump (10) to provide fresh medium supply to the culture when requested; it is electronically interfaced with the system. A switch valve (11) to switch between a medium supply and another. Medium supply bottles (12)-(13): containing the two types of medium supply used in this experiment (bottle size not in scale). Waste batch (14) to collect the PBR flowthrough. Adapted from the manufacturer hardware manual (https://psi.cz/). The illustration does not show the computer used to control the PBR and collect.

### **Supplementary Figure S2**

**
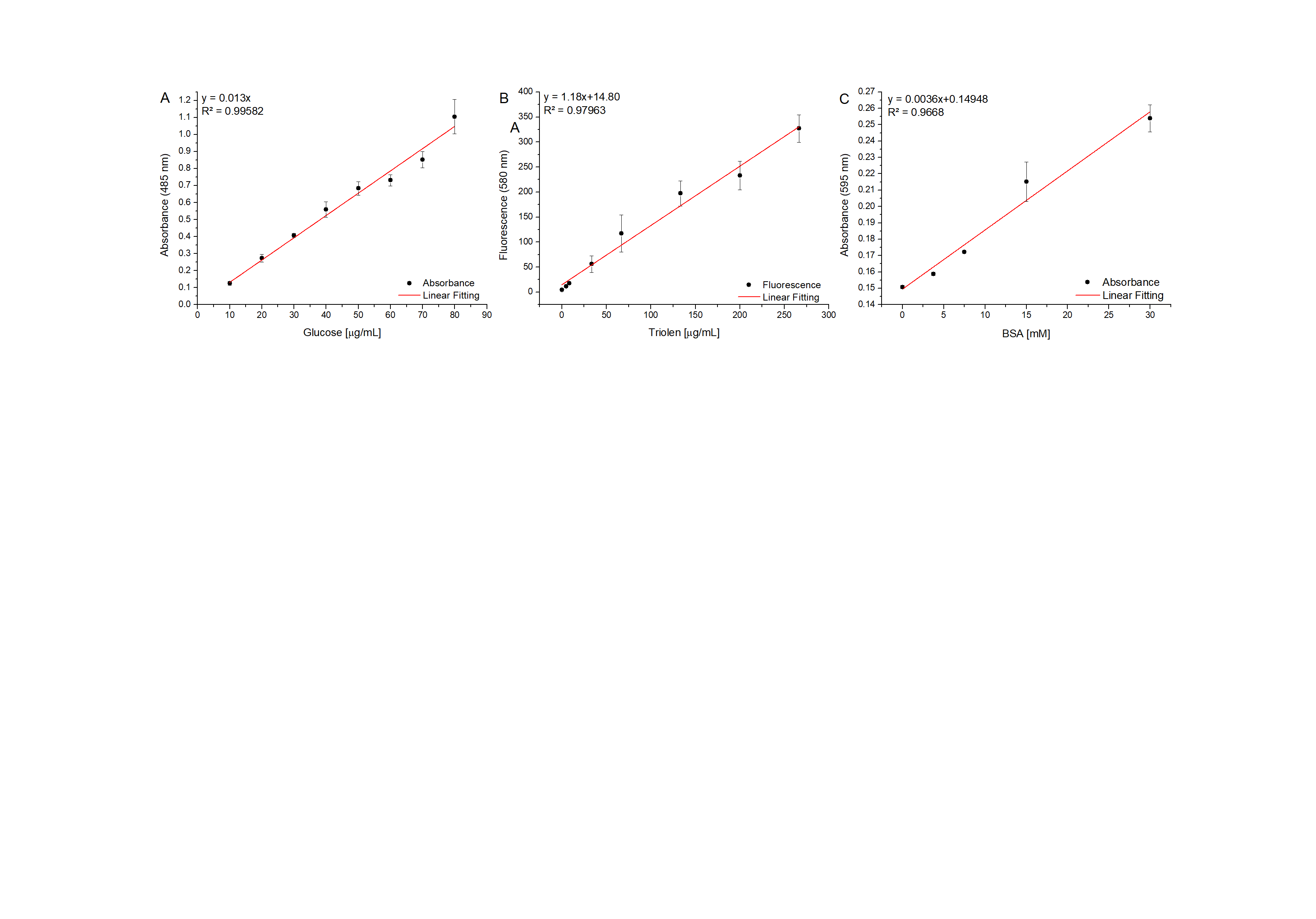
**

**Calibration curves for carbohydrates, lipids and proteins quantification.** (A) The calibration curve has been prepared with a sequential dilution of an aqueous solution of glucose. Total carbohydrate quantification has been done following the phenol-sulfuric acid method described by Nielsen (2017). (B) The calibration curve has been generated preparing a sequential dilution of glyceryl trioleate (Sigma-Aldrich T7140-500MG, purity ≥99%) in 25% DMSO (v/v in ultrapure water). Fluorescence spectra were recorded with a Perkin Elmer LS-55 (PerkinElmer^Ⓡ^, Excitation wavelength: 530nm, Emission: 545-800nm). (C) Calibration curve has been made with a serial dilution of bovine serum albumin (BSA, Sigma-Aldrich, purity ≥98%). Bradford method (Bradford, 1976) has been used for BSA quantification and xMark (Bio-Rad) spectrophotometer was used for the measurements.

### **Supplementary Figure S3**


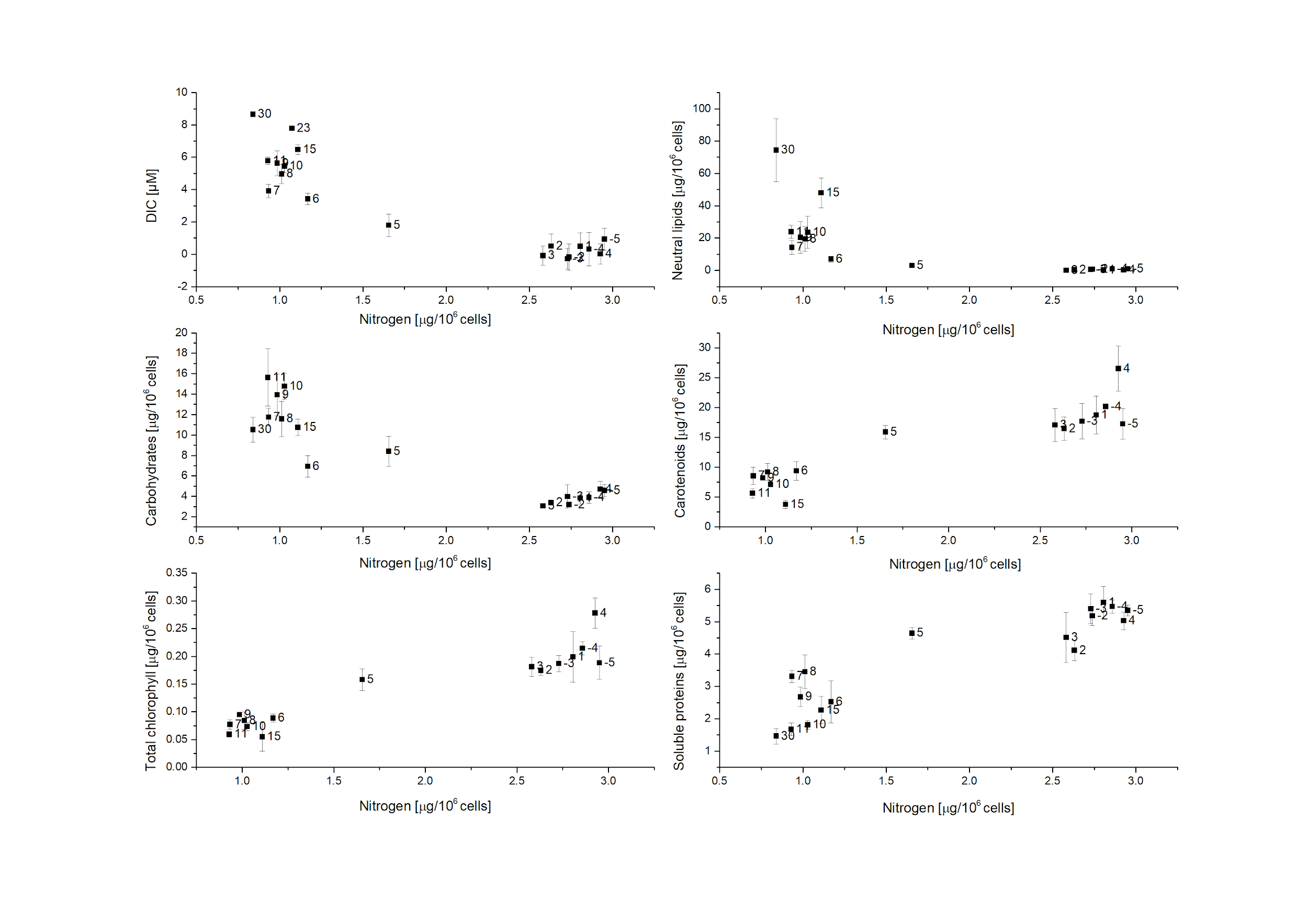


**Major organic macromolecules cellular quota and dissolved inorganic carbon in the culture medium according to the intracellular nitrogen.** Intracellular nitrogen (N-int) was considered as the parameter that guides the cell physiological and biochemical responses. Simple plots have been generated to observe the correlation between N-int and biochemical parameters. A similar pattern is observed in all the plots where an evolution during the time course of the experiment is observed. Two main groups are observed, with a transition on 5 and 6 DRNS. Each point has been marked with the respective time point after DNRS. All the graphs clearly show two major groups with the in between point corresponding to day 5 after DNRS, several times day 6 can be considered in between. All the information given by these graphs is grouped in the PCA (Fig.1).

### **Supplementary Figure S4**


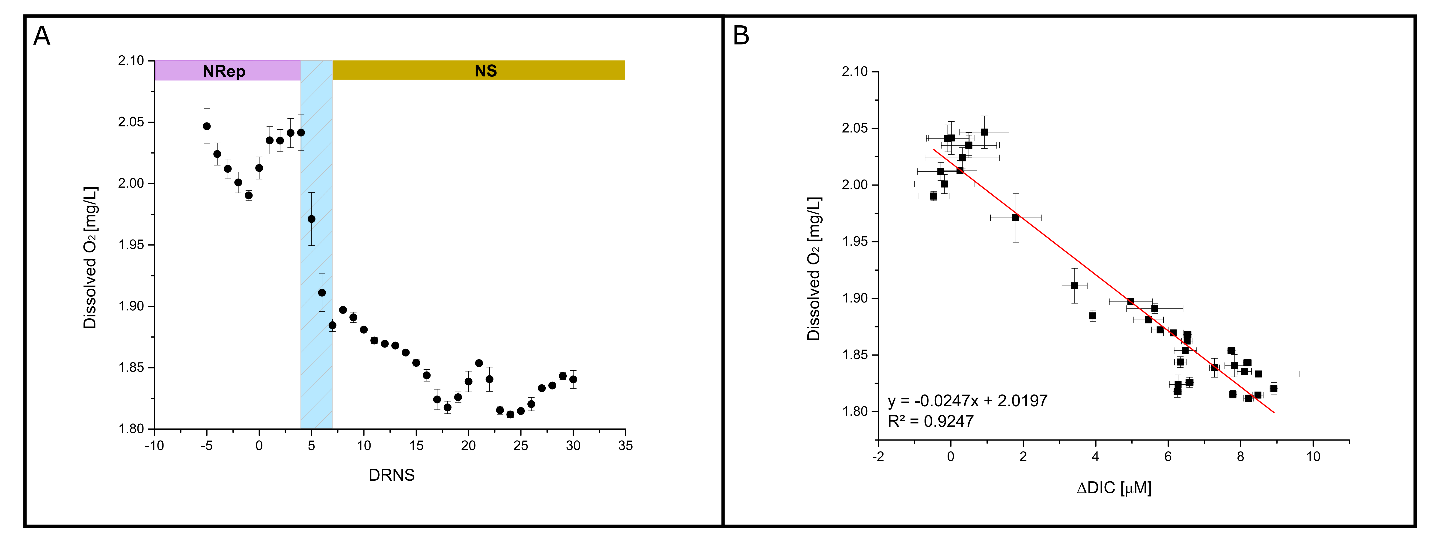


**Dissolved oxygen in the culture medium and its correlation with the change in DIC and photosynthetic parameters.** In photosynthetic organisms oxygen is both produced by photosynthesis and consumed by respiration. The oxygen input from the bubbling system being constant, the dissolved oxygen in the medium depends from the combined activity of respiration and photosynthetic. (A) Change in average daily dissolved oxygen during the time course of the experiment. The average value was calculated during 24h and omitting the period of dark exploited to relax the reaction centers of the photosystems. (B) Linear correlation between dissolved oxygen and change in DIC.

### **Supplementary Figure S5**


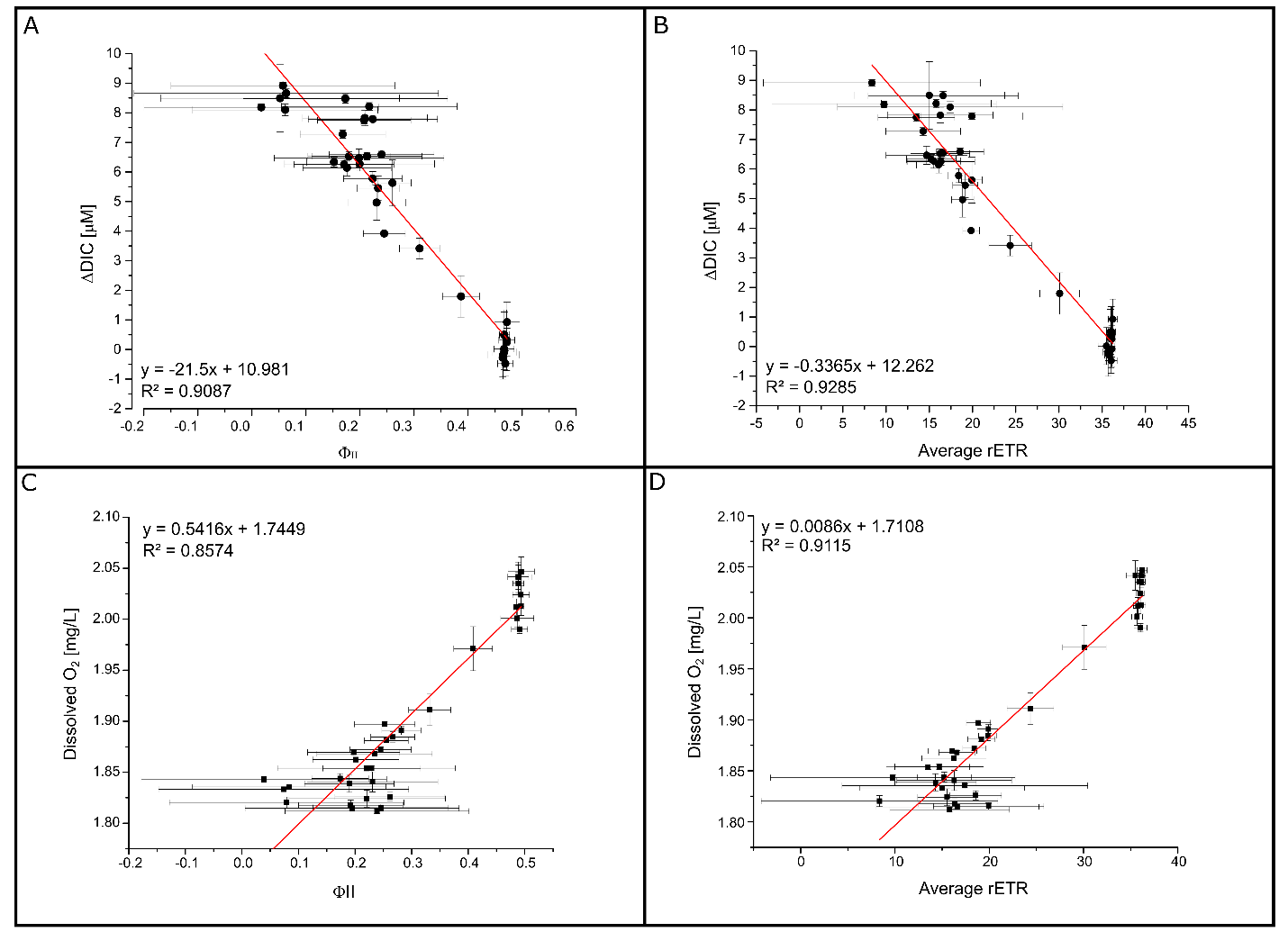


**Correlation between DIC, DIO and two photosynthetic activity parameters.** Photosynthetic organisms, including P. tricornutum, integrate inorganic carbon into organic molecules to the process of the photosynthesis. Changes in DIC and DIO may thus be correlated with the photosynthetic activity. To verify this statement linear correlations were performed between DIC and two different photosynthetic parameters. The first one is the maximum photochemical efficiency of PSII in LAS which serve as proxy to evaluate how much energy is directed trough the photochemistry and thus carbon fixation. The second one is the relative photosynthetic electron transport rate which gives information on the activity of the photosynthetic electron transport chain which generates energy exchange molecules later used by the CBB cycle. (A) Correlation between ΦII and the change in DIC. (B) Correlation between rETR and the change in DIC. (C) Linear correlation between dissolved oxygen and average daily rETR. (D) Linear correlation between dissolved oxygen and rETR.

### **Supplementary Figure S6**


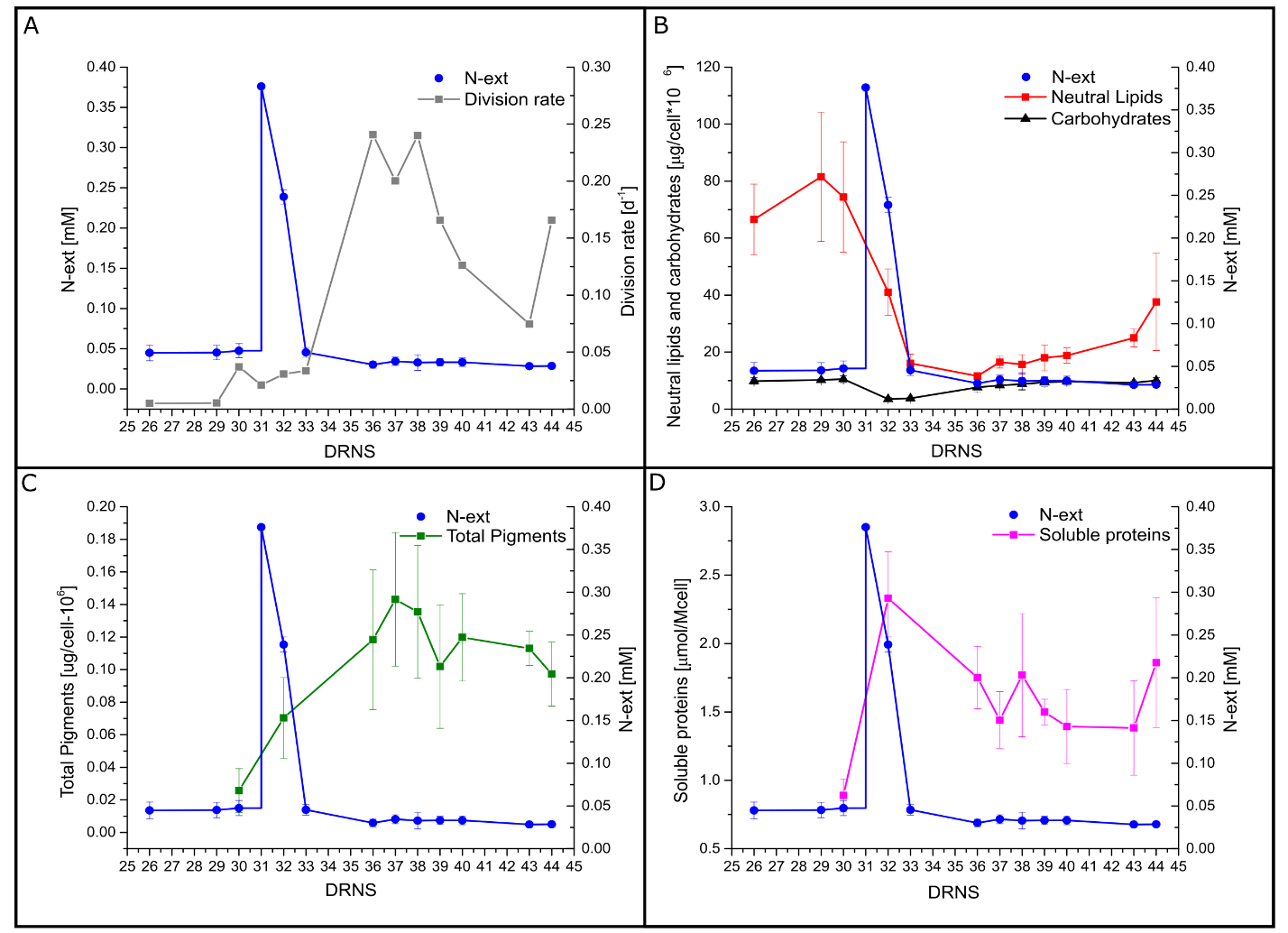


**Cell response to a small pulse of inorganic nitrogen.** After 31 days of nitrogen limited growing conditions, an amount of nitrate was introduced in the photobioreactor in order to increase the inorganic nitrogen availability to 0.37 mM. The cells directly reacted to the increased nitrogen availability The biochemical a physiological response have been recorded and results are reported in the following figure. (A) Cell division rate response to the nitrogen pulse. (B) Change in carbohydrate and neutral lipid cell content in response to the nitrogen pulse. (C) Change in total pigment cell content in response to the nitrogen pulse. (D) Change in soluble protein cell content in response to the nitrogen pulse.

### **Supplementary Figure S7**


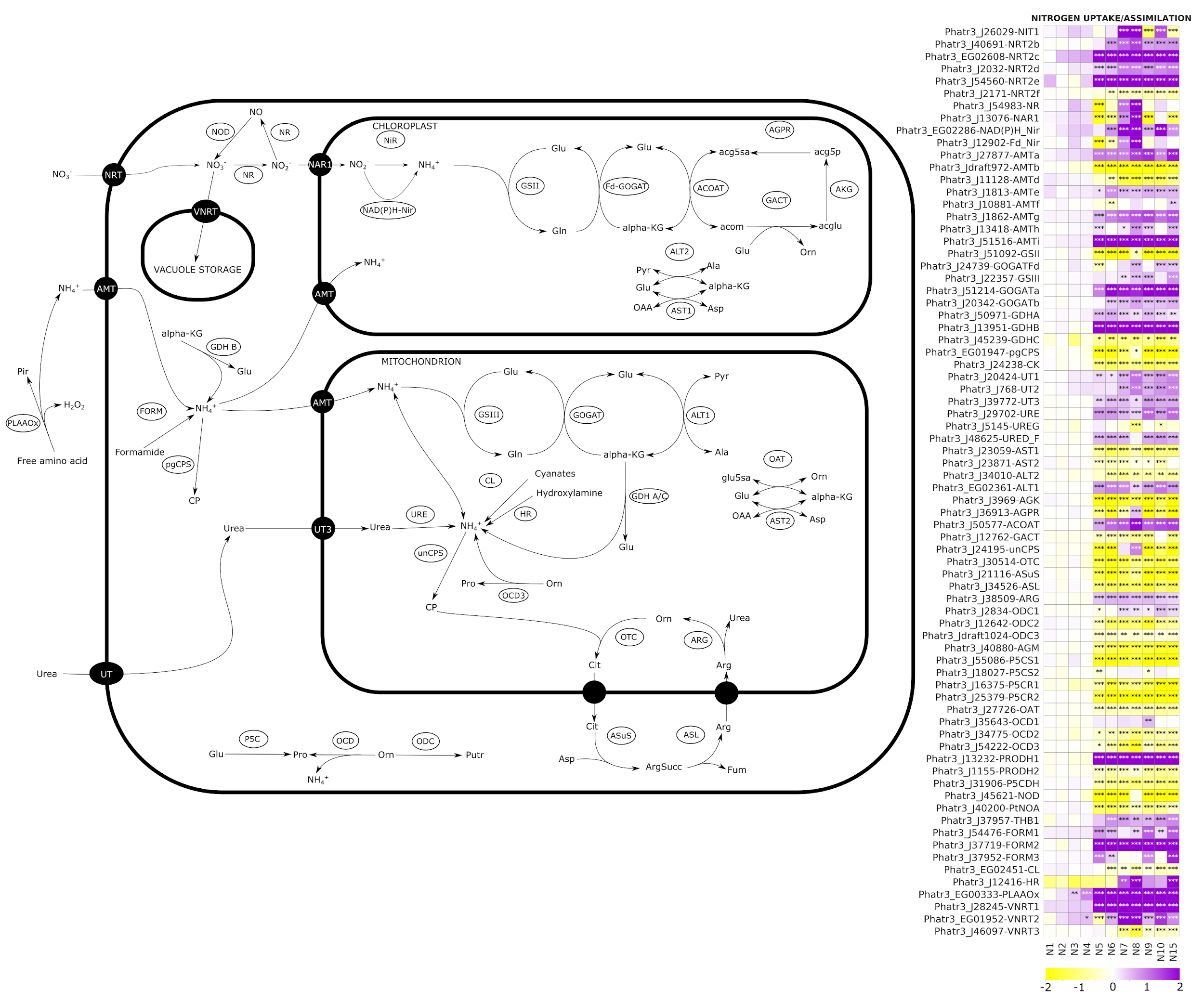


**Nitrogen uptake and assimilation pathway in P. tricornutum with detailed daily heatmap showing the change in gene expression during the time course of the experiment.** NRT, nitrate transporter; VNRT, vacuolar nitrate transporter; NR, nitrate reductase; NAR1, nitrite transporter; Nir, nitrite reductase; NAD(P)H-Nir, nitrite reductase NAD(P)H dependent; GSII/III, glutamate-ammonia ligase; GOGAT, glutamate synthase; ACOAT, acetylornithine aminotransferase; AGPR, N-acetyl-gamma-glutamyl-phosphate reductase; GACT, glutamate N-acetyltransferase; AKG, acetylglutamate kinase; ALT, L-alanine transaminase; AST, aspartate aminotransferase; PLAAOx, periplasmic L-amino acid oxidase; FORM, formamidase; GDH, glutamate dehydrogenase; CP, carbamoyl phosphate; pgCPS, carbamoyl phosphate synthetase; unCPS, carbamoyl phosphate synthase mitochondrial, UT, urea transporter; URE, urease; CL, cyanate lyase, HR, Hydroxylamine reductase; GDH, glutamate dehydrogenase; OCD, ornithine cyclodeaminase; ODC, ornithine decarboxylase; P5CS, pyrroline-5-carboxylate synthase; P5CR, pyrroline-5-carboxylate reductase; OTC, ornithine transcarbamylase; ARG, arginase; ASL, argininosuccinate lyase; ASuS, argininosuccinate hydratase

### **Supplementary Figure S8**


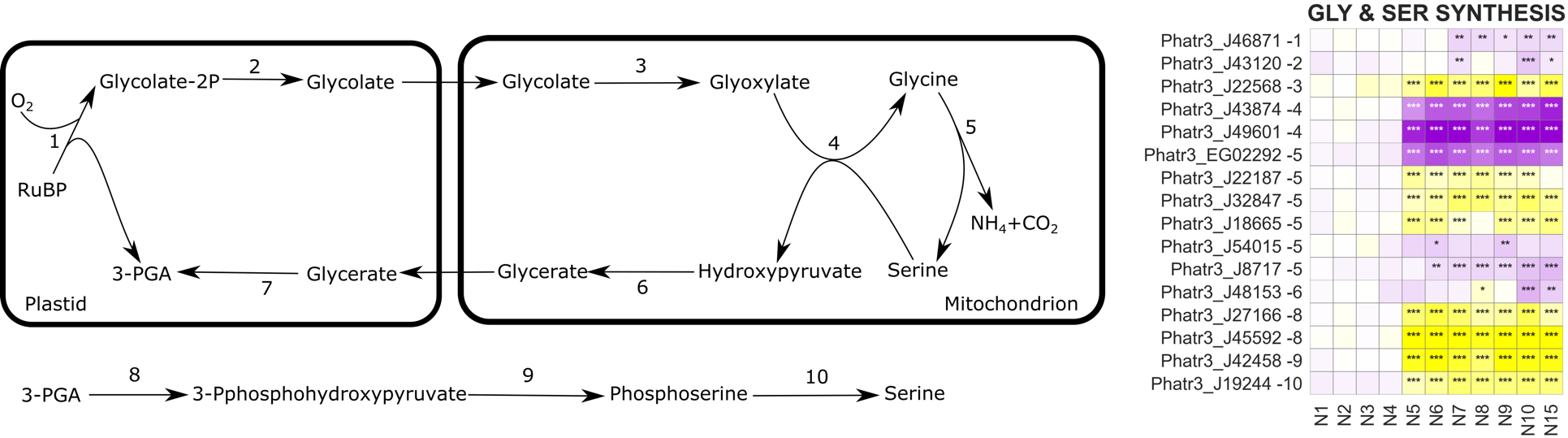


**Serine (SER) and glycine (GLY) biosynthesis.** RuBP, Ribulose 1,5-bisphosphate.

### **Supplementary Figure S9**


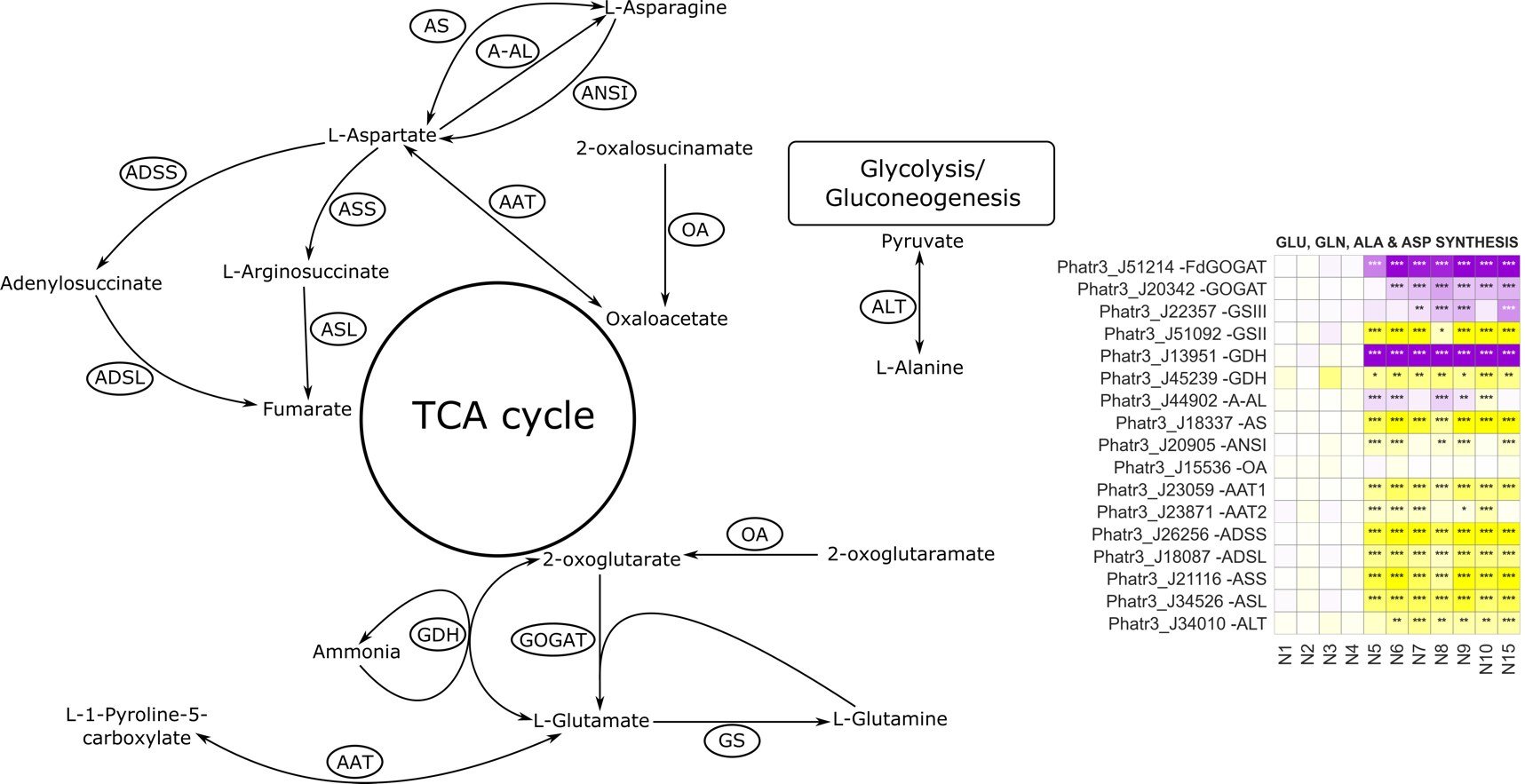


**Glutamate (GLU), glutamine (GLN), alanine (ALA) and aspartate (ASP) biosynthesis.** GOGAT, Glutamate synthase; GS, glutamine synthase; GDH, glutamate dehydrogenase; A-AL, aspartate ammonia ligase; AS, asparagine synthase; ANSI, asparaginase; OA, omega-amidase; AAT, aspartate transaminase; ADSS, Adenylosuccinate synthetase; ADSL, Adenylosuccinate lyase; ASS, citrulline-aspartate ligase; ASL, arginosuccinate lyase; ALT, Alanine transaminase.

### **Supplementary Figure S10**


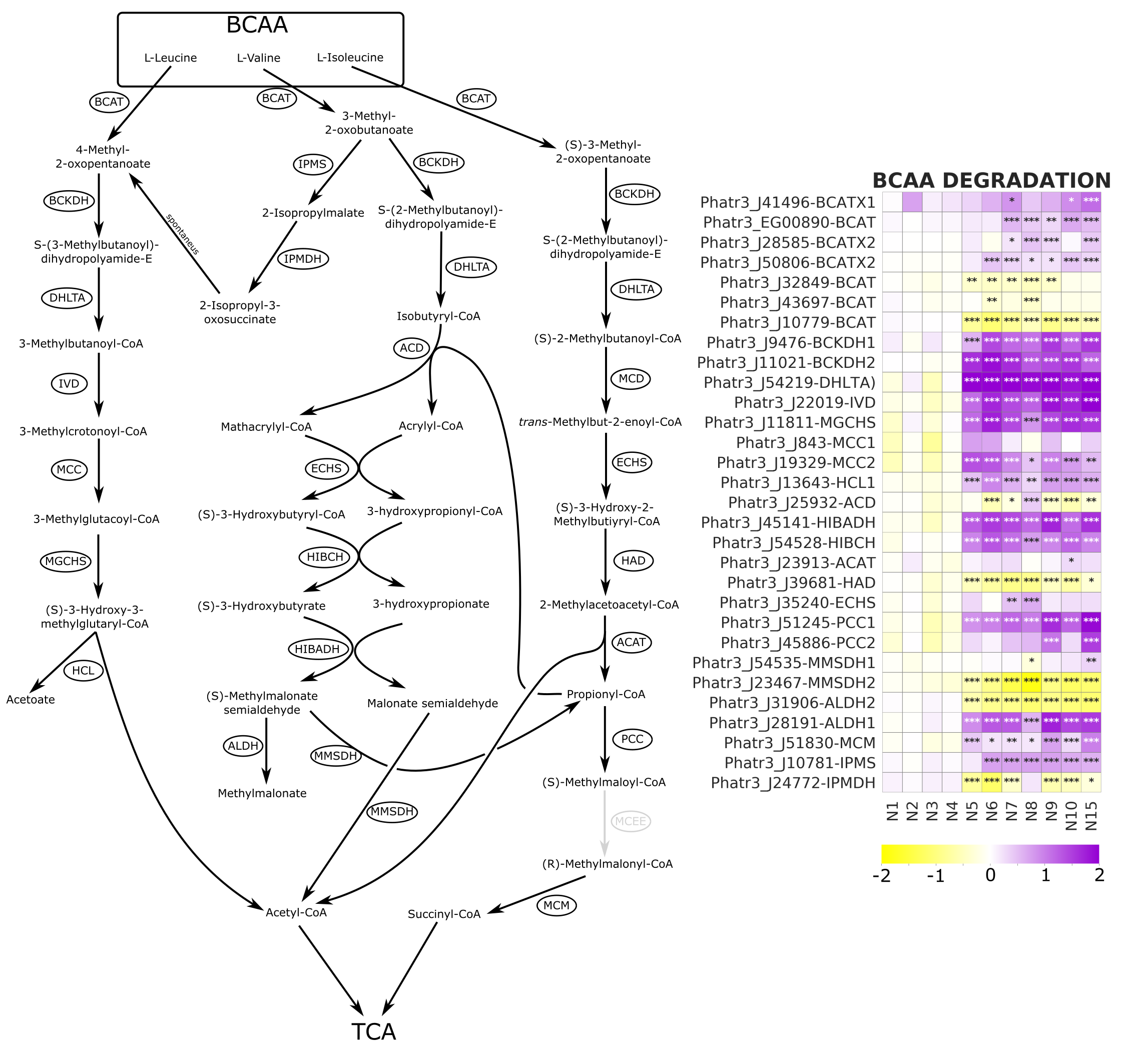


**Pathways of BCAA degradation in P. tricornutum with the heatmap reporting the change in gene expression change during the time course of the experiment.** Modified from Pan et al., 2018. BCAT, branched-chain amino acid transaminase; BCKDH, branched-chain a-keto acid dehydrogenase; DHLTA, dihydrolipoyllysine-residue (2-methylpropanoyl) transferase; IVD, isovaleryl-CoA dehydro- genase; MCC, methylcrotonyl-CoA carboxylase; MGCHS, methylglutaconyl-CoA hydratase; HCL, hydroxymethylglutaryl-CoA lyase; IPMS, 2-isopropylmalate synthase; IPMDH, isopropylmalate dehydratase; IPMDCase, 3-isopropylmalate dehydrogenase; MCD, 2-methylacyl-CoA dehydrogenase; ECHS, enoyl-CoA hydratase; HAD, 3- hydroxyacyl-CoA dehydrogenase; ACAT, acetyl-CoA C-acyltransferase; ACD, acyl-CoA dehydrogenase; HIBCH, 3-hydroxyisobutyryl-CoA hydrolase; HIBADH, 3-hydroxyi- sobutyrate dehydrogenase; MMSDH, methylmalonate-semialdehyde dehydrogenase; ALDH, aldehyde dehydrogenase; PCC, propionyl-CoA carboxylase; MCM, methylmalonyl-CoA mutase; MCEE, methylmalonyl-CoA epimerase.

### **Supplementary Figure S11**


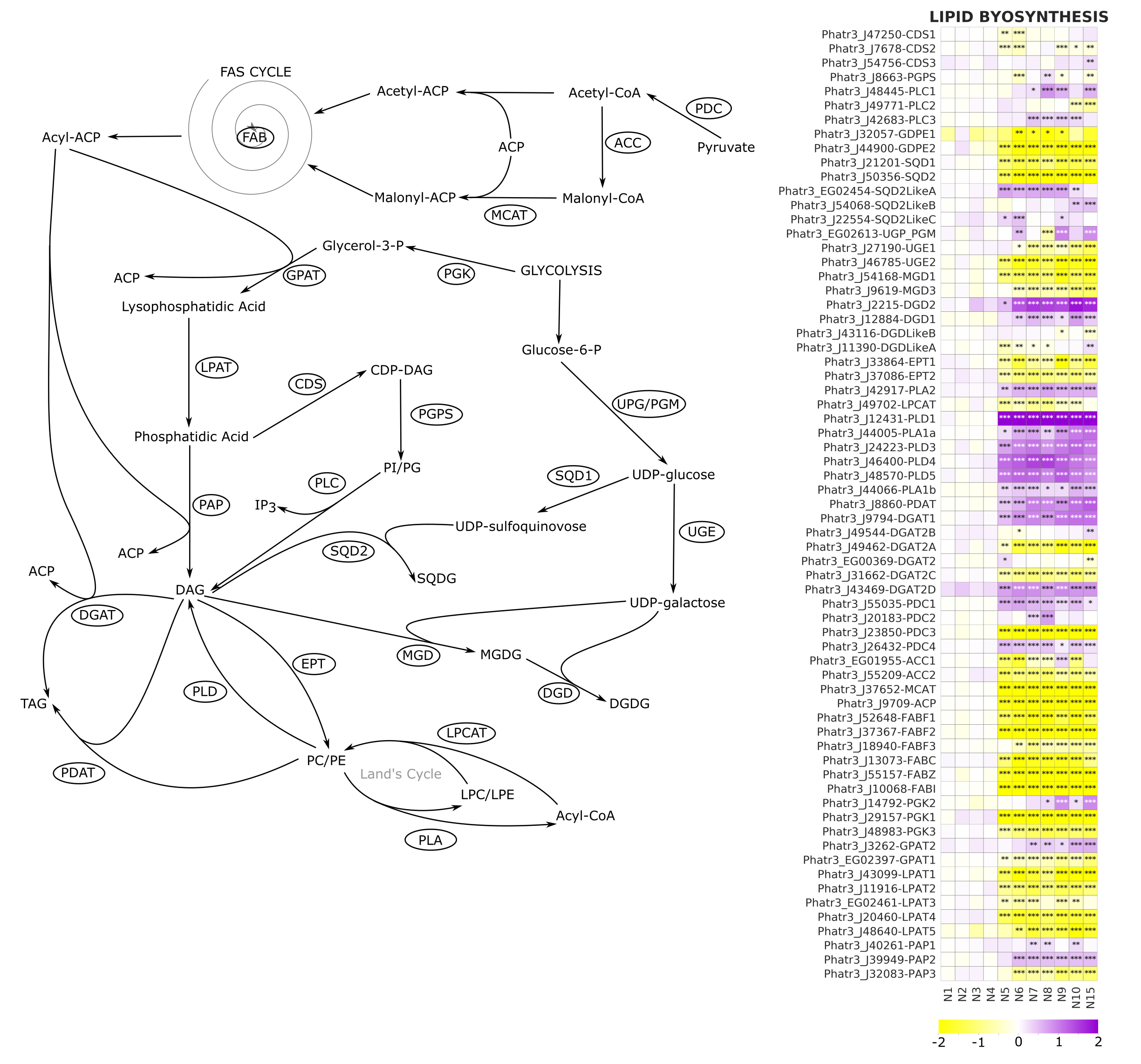


**Lipid biosynthesis and remodeling pathways in P. tricornutum as reported in the main text.** The heatmap reports the daily change in gene expression during the time course of the experiment. PDC, pyruvate dehydrogenase complex; ACC, acetyl-CoA carboxylase; MCAT, malonyl-CoA:ACP transacylase; FAB, fatty acid biosynthesis; PGK, phosphoglycerate kinase; GPAT, glycerol-3-phosphate: acyl-ACP acyltransferase; LPAT, lysophosphatidate acyltransferase; PAP, phosphatidate phosphatase; CDS, phosphatidate cytidylyltransferase; PGPS, CDP-diacylglycerol--glycerol-3-phosphate 3-phosphatidyltransferase, PLC, phospholipase C; SQD1, UDP-sulfoquinovose synthase; SQD2, glycosyl transferase; UPG/PGM, UDP-glucose-pyrophosphorylase/phosphoglucomutase; UGE, UDP-galactose 4-epimerase; MGD, MGDG synthase; DGD, DGDG synthase; EPT, ethanolamine phosphotransferase; PLD, phospholipase D; PLA, phospholipase A; LPCAT, lysophosphatidylcholine acyltransferase; PDAT, phospholipid:diacylglycerol acyltransferase; DGAT, acyl CoA:diacylglycerol acyltransferase; ACP, acyl carrier protein.

### **Supplementary Figure S12**


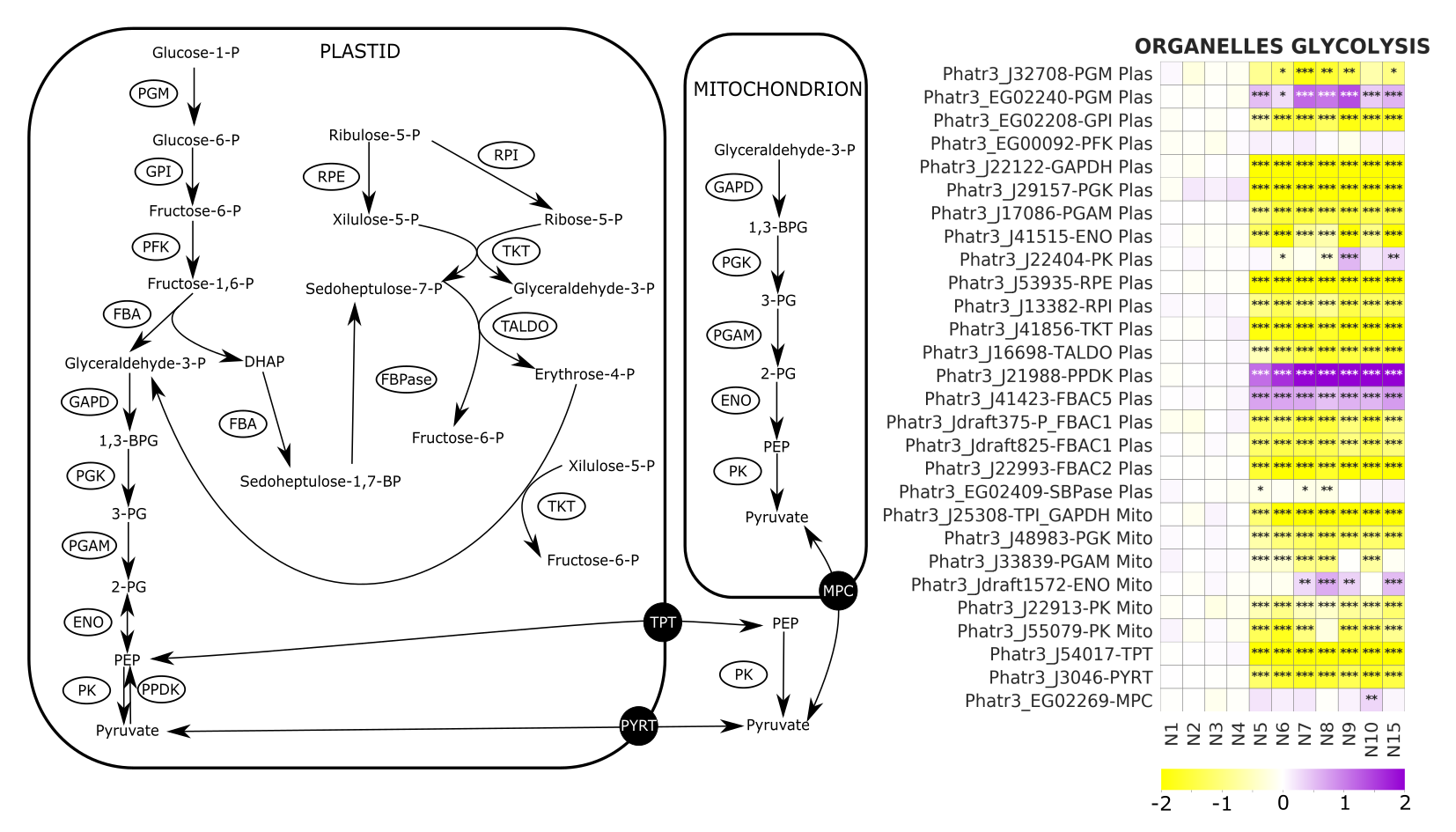


**Detailed glycolytic pathways heatmap reporting the detailed gene expression change in the two organelles.** PGM, phosphoglucomutase; GPI, glucose-6-phosphate isomerase; PFK, phosphofructokinase; FBA, fructose bisphosphate aldolase; GAPD, glyceraldehyde-3-phosphate dehydrogenase; PGK, phosphoglycerate kinase; PGAM, phosphoglycerate mutase; ENO, enolase; PK, pyruvate kinase; PPDK, Pyruvate, phosphate dikinase; FBP, fructose-1,6-bisphosphatase; RPE2, ribulose-phosphate 3-epimerase; TKT, transketolase; RPI, ribose-5-phosphate isomerase; ALDO, transaldolase; TPT, plastidic triose-phosphate/phosphate translocator, PYRT, pyruvate transporters; MPC, mitochondrial pyruvate carrier.

### **Supplementary Figure S13**


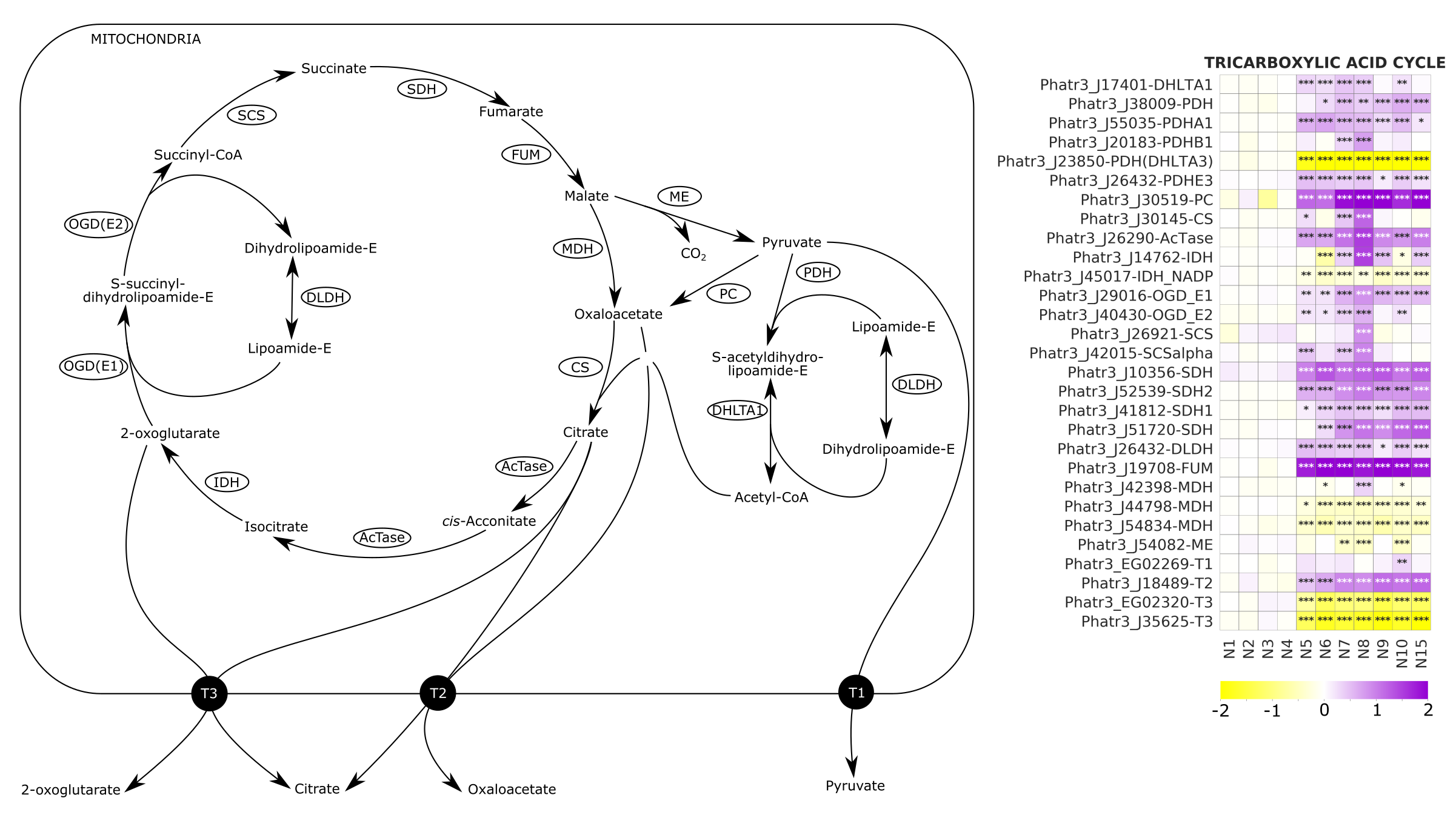


**Tricarboxylic acid cycle with detailed daily heatmap showing the change in gene expression during the time course of the experiment.** PC, pyruvate carboxylase; ME, malic enzyme; PDH, pyruvate dehydrogenase; DLDH, dihydrolipoyl dehydrogenase; DHLTA, dihydrolipoamide s-acetyltransferase; MDH, malate dehydrogenase; CS, citrate synthase; AcTase, aconitase; IDH, isocitrate dehydrogenase; OGD, oxoglutarate dehydrogenase; SCS, succinate CoA ligase; SDH, succinate dehydrogenase; FUM, fumarase.

### **Supplementary Figure S14**


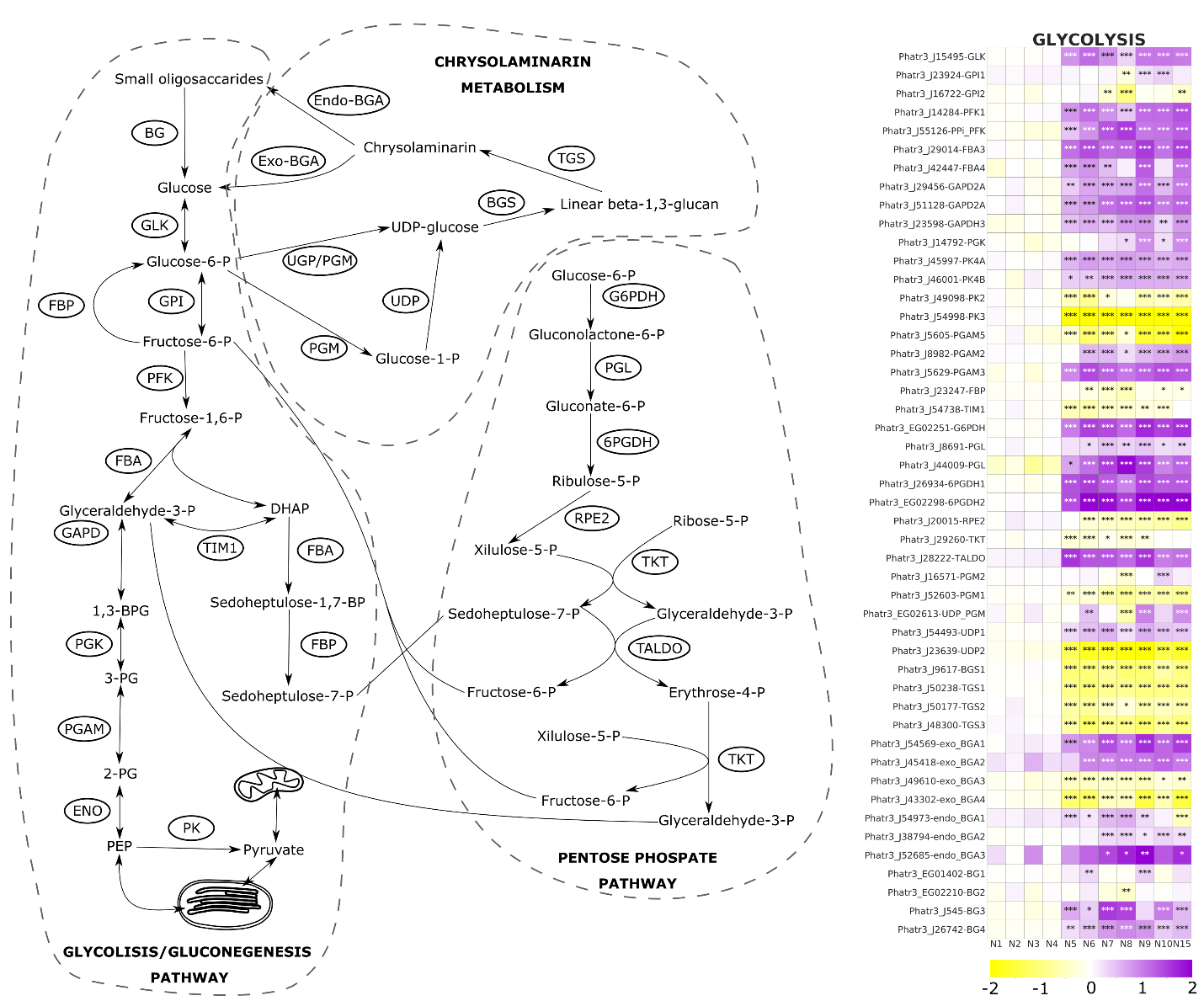


**Glycolysis/gluconeogenesis, pentose phosphate pathway and chrysolaminarin metabolism in P.tricornutum with the detailed daily heatmap reporting the change in gene expression during the time course of the experiment.** BG, beta-glucosidase, GLK, glucokinase; GPI, glucose-6-phosphate isomerase; FBP, fructose-1,6-bisphosphatase; PFK, phosphofructokinase; FBA, fructose bisphosphate aldolase; GAPD, glyceraldehyde-3-phosphate dehydrogenase; PGK, phosphoglycerate kinase; PGAM, phosphoglycerate mutase; ENO, enolase; PK, pyruvate kinase; TIM, triosephosphate isomerase; G6PDH, glucose 6-phosphate dehydrogenase; PGL, 6-phosphogluconolactonase, 6PGDH, phosphogluconate dehydrogenase; RPE2, ribulose-phosphate 3-epimerase; TKT, transketolase; TALDO, transaldolase; UPG/PGM, UDP-glucose-pyrophosphorylase/phosphoglucomutase; PGM, phosphoglucomutase; UGP, UTP--glucose-1-phosphate uridylyltransferase; BGS, 1,3-beta-glucan synthase; TGS, 1,6-β-transglycosylases; Exo-BGA, exo-beta-glucanase; Endo-BGA, endo-beta-glucanase.

### **Supplementary Table S1**

**List of publications reporting information on nitrogen starvation response in P. tricornutum.** (a) P. tricornutum culture collection ID and ecotype, information is reported according to each reference. (b) Type of treatment that has been performed. N- treatment refers to an exposure of the P. tricornutum strain to N starvation; N+ to N- comparison refers to the comparisons between N availability and N limitation; N+ to N- transfer refers to a transfer of cells from nitrogen replated to nitrogen starved conditions; N+ to N- transition refers to a transition from N repleted to N starved conditions. Comparisons between different nitrogen sources and N and P combined limitation are specified. (c) Different nitrogen concentration used for N+ and N- treatment reported in the corresponding reference. (d) Type of culture used in the reference publication: batch in flask of photobioreactor (PBR) or continuous in PBR (chemostat or turbidostat are specified). (e) Type of medium used: ASP-2 (Provasoli et al., 1957); f/2 medium (Guillard and Ryther, 1962); ASW, artificial sea water; ESAW, enriched ASW; RSE medium. Particular medium types and variants is specified. Walne solution (Walne, 1966). For f/4 medium composition refer to the respective publication. (f) Light cycle and light intensity, not always the information is available. (g) Temperature, not always the information is available. (h) Carbon source. Mainly through air bubbling, air flux and CO_2_ percentage are reported when available (CO_2_ in the air was 409.8 ppm in 2019). The table is not exhaustive and may omit some publications and relative information.

| **Reference** | **(a)**  **Strain (ecotype)** | **(b)**  **Type of N- treatment** | **(c)**  **N-ext concentration when starved** | **(d)**  **Type of culture** | **(e)**  **Type of medium** | **(f)**  **Light cycle**  **(Intensity μmoles of photons m^-2^s^-1^)** | **(g)**  **Temperature** | **(h)**  **Carbon source** |
| --- | --- | --- | --- | --- | --- | --- | --- | --- |
| Larson and Rees (1996) | CCAP1052/6 (Pt4) | N+ and N-  comparison | - 10 mM (N+)  - 0 mM (N-) | Batch  (Flask) | ASP-2 | N/A  Not light limited | N/A | Natural diffusion |
| Valenzuela *et al.* (2012) | CCMP2561  (Pt1) | N+/P+ to N-/P- transition | From 580 μM to  0 μM | Batch  (PBR) | ASP-2 | 14:10  (450) | 20°C | 0.40 L min^-1^ Air bubbling |
| Yang *et al.* (2013) | FACHB-863  (?) | N+ to N-  Transfer | - 75 mg/L (N+)  - 0 mg/L (N-) | Batch  (Flask) | f/2 - Si | 12:12  (200) | 21°C | Natural diffusion |
| Ge *et al.* (2014) | CCMP2561  (Pt1) | N+ to N-  Transfer | - 300 μM  - 500 μM  - 700 μM | Batch | f/2 | Continuous  (100) | 22°C | Air bubbling |
| Levitan *et al.* (2015a) | Strain 8.6  (Pt1) | N+ and N-  comparison | - 880 μM (N+)  - 0 μM (N-) | Batch  (Flask) | ASW + f/2 nutrients | Continuous  (120–150) | 18°C | Air bubbling |
| Levitan *et al.* (2015b) | Strain 8.6  (Pt1) | N+ and N-  comparison | - 880 μM (N+)  - 0 μM (N-) | Batch  (Flask) | ASW + f/2 nutrients | Continuous  (120–150) | 18°C | Air bubbling |
| Abida *et al.* (2015) | 1055/3  (Pt1) | N+ and N-  comparison | - 550 μM (N+)  - 0 μM (N-) | Batch  (Flask) | ESAW | 12:12  (200) | 19°C | Natural diffusion |
| Alipanah *et al.* (2015) | CCMP632  (Pt1) | N+ and N-  comparison | - 909 μM (N+)  - 10 μM (N-) | Batch  (Flask) | f/2 | Continuous  (60) | 15°C | Natural diffusion |
| Zhao *et al.* (2015) | UTEX 640  (Pt4) | N+ and N- comparison | - 440 μM (N+)  - 220 μM (N- #1)  - 110 μM (N- #2) | Batch | f/4 | 12:12  (100-150) | 19°C | Natural  diffusion |
| Longworth *et al.* (2016) | CCAP1055/1  (Pt1) | N+ and N-  comparison | - 882 μM (N+)  - 88.2 μM (N-) | Batch  Column PBR | f/2 + Si | Continuous  (200) | 25°C | Air bubbling |
| Popko *et al.* (2016) | UTEX 646  (Pt4) | N+ and N-  comparison | - 18.8 mM (N+)  - 0 mM (N-) | Fed batch  Column PBR | RSE | Continuous  N+ (120)  N- (170/350) | N/A | Air bubbling  (2% CO_2_) |
| Matthijs *et al.* (2016) | Strain 8.6  (Pt1) | N+ and N-  comparison | - 7.5 mg L^-1^ (N+)  - 0 mg L^-1^ (N-) | Batch  (Flask) | ESAW | Continuous  (100) | 21°C | Natural diffusion |
| Matthijs *et al.* (2017) | Strain 8.6  (Pt1) | N+ and N-  comparison | - 7.5 mg L^-1^ (N+)  - 0 mg L^-1^ (N-) | Batch  (Algem PBR, Algenuity, UK) | ESAW | Continuous  (150) | 21°C | Natural diffusion |
| Yodsuwan *et al.* (2017) | National  Research Institute of Aquaculture, Minami-Ise, Japan (?) | N+ and N-  comparison | - 0 mg L^-1^  -16.45 mg L^-1^  - 32.09 mg L^-1^  - 64.29 mg L^-1^ | Batch  (Flask) | Sea water + f/2 nutrients | 14:10  (80-100) | 20°C | Natural diffusion |
| Remmers *et al.* (2017) | SAG1090-1b  (Pt3) | N- treatment | - 16.8 mM (Prior treatment)  - 5 mM (Treatment) | Continuous chemostat  (Flat panel PBR) | Custom | 16:8  (160->260) | 20°C | 0.4 L min^-1^ Air bubbling  (2% CO_2_) |
| Remmers *et al.* (2018) | SAG1090-1b  (Pt3) | N+ and N- comparison | - 0.11 gN d^−1^ (N+)  - 0.02 gN d^−1^(N-) | Continuous turbidostat  (Flat panel PBR) | Custom | 16:8  (180) | 20°C | 1.7 L min^-1^ Air bubbling  (1% CO_2_) |
| Huang *et al.* (2019) | UTEX 646 (Pt4) | N+ and N- comparison | - 1.176 mM (N+)  - 0.125 mM (N-) | Batch  (Flask) | Seawater + Walne solution | Continuous  (300) | 22°C | Air bubbling |
| Wang *et al.* (2019) | CCMP 2561  (Pt1) | N+ and N- comparison | - 0.882 mM (N+)  - 0.0882 mM (N+) | Batch and semi continuous  (Flask) | f/2 | Continuous  (150) | 20°C | Air bubbling  (650 ppm CO_2_) |
| Smith *et al.* (2019) | CCAP 1055  (?) | NO_3_^-^, NO_2_^-^, NH_4_^+^ and N- comparison | - 0 mM (N-)  - 0.3 mM (N sources) | Batch  (Flask) | ASW + f/2 nutrients | 14:10  (150) | 18°C | Air bubbling |

### **Supplementary Table S2**

**Common fluorescence parameters**. Used throughout the text for quantification of the relative electron transport rate (rETR) as well as the photochemical (ΦP0, qP, ΦP, ΦII) and non-photochemical (qN, NPQ) processes (Equations according to Roháček et al. (2008)

| **Symbol** | **Name** | **Description** |
| --- | --- | --- |
| rETR | Relative electron transport rate | rETR=((F_m_-F_t_)/F_m_)×E×0.84*×0.5*  Relative photosynthetic electron transport rate. E refers to the intensity of photosynthetically active radiation (Genty *et al.*, 1989)) |
| F_0_ | Instantaneous fluorescence in DAS | Instantaneous fluorescence yield in dark |
| F_s_ | Instantaneous fluorescence in LAS | Instantaneous fluorescence yield when a dynamic equilibrium of plastoquinone reducing, re-oxidizing processes and from non-photochemical quenching is established |
| F_M_ | Maximum fluorescence in DAS | Maximum fluorescence yield with Q_A_ reduced and non-photochemical quenching relaxed |
| F_M_’ | Maximum fluorescence in LAS | Maximum fluorescence yield with Q_A_ reduced and non-photochemical quenching active |
| F_V_ | Variable fluorescence | F_V_ = F_M_ - F_0_ |
| Φ_P0_ | Instantaneous PSII quantum yield in DAS | Quantifies the maximum photochemical efficiency of PSII in DAS and serves as a proxy of the fitness of the photosynthetic apparatus.  Φ_P0_=F_V_/F_M_ |
| Φ_II_ | Instantaneous PSII quantum yield in LAS | Quantifies the maximum photochemical efficiency of PSII in LAS and serves as a proxy of the fitness of the photosynthetic apparatus.  Φ_II_= (F_M_' – F_S_)/F_M_' |
| qP | Photochemical quenching of variable Chl fluorescence | Parameter estimating the fraction of open PS II centers  qP = (F_M_' – F_S_)/(F_M_' – F_0_') = ∆F/F_V_ |
| qN | Non-photochemical quenching of variable Chl fluorescence | Indicates the amount of energy that is quenched through heat dissipation  qN = (F_V_ – F_V_')/F_V_ = 1 – F_V_'/F_V_ |
| NPQ | Non-photochemical Chl fluorescence quenching | Indicates the amount of energy that is quenched through heat dissipation  NPQ = (F_M_ – F_M_’)/F_M_' = F_M_/F_M_' – 1 |

### **Supplementary Table S3**

**Experiment sampling program.** In order to perform the required analyses to have insight on biochemical and molecular responses of P. tricornutum to the transition from N repleted to N limited conditions, culture samples have been collected throughout the experiment time course. The following table summarizes the sampling program, reporting the sampling volume for each type of analyses and the quantity of sample replicates.

| **Days After DRNS** | **RNA** | | **Metabolites** | | **C and N quantification** | **Carbohydrates** | **Lipids** | **Pigments** | **Proteins** | **Inorganic N in the medium** |
| --- | --- | --- | --- | --- | --- | --- | --- | --- | --- | --- |
|  | # | Volume [mL] | # | Volume [mL] | 6mL | 2mL | 2mL | 2mL | 2mL | 2mL |
| -5 | 3X | 20 | 2X | 10 | X | 3X | 3X | 3X | 3X | 3X |
| -4 | 3X | 20 | 2X | 10 | X | 3X | 3X | 3X | 3X | 3X |
| -3 | 3X | 20 | 2X | 10 | X | 3X | 3X | 3X | 3X | 3X |
| -2 | 3X | 20 | 2X | 10 | X | 3X | 3X | 3X | 3X | 3X |
| 1 | 3X | 20 | 2X | 10 | X | 3X | 3X | 3X | 3X | 3X |
| 2 | 3X | 20 | 2X | 10 | X | 3X | 3X | 3X | 3X | 3X |
| 3 | 3X | 20 | 2X | 10 | X | 3X | 3X | 3X | 3X | 3X |
| 4 | 3X | 20 | 2X | 10 | X | 3X | 3X | 3X | 3X | 3X |
| 5 | 3X | 20 | 2X | 10 | X | 3X | 3X | 3X | 3X | 3X |
| 6 | 3X | 15 | 2X | 10 | X | 3X | 3X | 3X | 3X | 3X |
| 7 | 3X | 15 | 2X | 10 | X | 3X | 3X | 3X | 3X | 3X |
| 8 | 3X | 15 | 2X | 10 | X | 3X | 3X | 3X | 3X | 3X |
| 9 | 3X | 15 | 2X | 10 | X | 3X | 3X | 3X | 3X | 3X |
| 10 | 2X | 15 | 2X | 10 | X | 3X | 3X | 3X | 3X | 3X |
| 11 | 2X | 15 | 2X | 10 | X | 3X | 3X | 3X | 3X | 3X |
| 15 | 2X | 15 |  |  | X | 3X | 3X | 3X | 3X | 3X |
| 16 |  |  |  |  |  | 3X | 3X |  |  |  |
| 17 |  |  |  |  |  | 3X | 3X |  |  |  |
| 18 |  |  |  |  |  | 3X | 3X |  |  |  |
| 19 |  |  |  |  |  | 3X | 3X |  |  |  |
| 20 |  |  |  |  |  | 3X | 3X |  |  |  |
| 22 |  |  |  |  |  | 3X | 3X |  |  |  |
| 23 |  |  |  |  | X |  |  |  |  |  |
| 26 |  |  |  |  |  | 3X | 3X |  |  |  |
| 29 |  |  |  |  |  | 3X | 3X |  |  |  |
| 30 |  |  |  |  | X | 3X | 3X | 3X | 3X | 3X |

**References**

**Abida H, Dolch LJ, Mei C, Villanova V, Conte M, Block MA, Finazzi G, Bastien O, Tirichine L, Bowler C, Rebeille F, Petroutsos D, Jouhet J, Marechal E**. 2015. Membrane glycerolipid remodeling triggered by nitrogen and phosphorus starvation in *Phaeodactylum tricornutum*. Plant Physiology **167**, 118-136.

**Alipanah L, Rohloff J, Winge P, Bones AM, Brembu T**. 2015. Whole-cell response to nitrogen deprivation in the diatom *Phaeodactylum tricornutum*. Journal of Experimental Botany **66**, 6281-6296.

**Bradford MM**. 1976. A rapid and sensitive method for the quantitation of microgram quantities of protein utilizing the principle of protein-dye binding. Analytical Biochemistry **72**, 248-254.

**Ge F, Huang W, Chen Z, Zhang C, Xiong Q, Bowler C, Yang J, Xu J, Hu H**. 2014. Methylcrotonyl-CoA carboxylase regulates triacylglycerol accumulation in the model diatom *Phaeodactylum tricornutum*. Plant Cell **26**, 1681-1697.

**Genty B, Briantais JM, Baker NR**. 1989. The relationship between the quantum yield of photosynthetic electron transport and quenching of chlorophyll fluorescence. Biochimica et Biophysica Acta **990**, 87-92.

**Guillard RR, Ryther JH**. 1962. Studies of marine planktonic diatoms: I. *Cyclotella nana* and *Detonula confervacea* (Cleve). CANADIAN JOURNAL OF MICROBIOLOGY **8**, 229-239.

**Huang B, Marchand J, Blanckaert V, Lukomska E, Ulmann L, Wielgosz-Collin G, Rabesaotra V, Moreau B, Bougaran G, Mimouni V, Morant-Manceau A**. 2019. Nitrogen and phosphorus limitations induce carbon partitioning and membrane lipid remodelling in the marine diatom *Phaeodactylum tricornutum*. European Journal of Phycology, 1-17.

**Larson TR, Rees TAV**. 1996. Changes in cell composition and lipid metabolism mediated by sodium and nitrogen availability in the marine diatom *Phaeodactylum tricornutum* (Bacillariophyceae). Journal of Phycology **32**, 388-393.

**Levitan O, Dinamarca J, Zelzion E, Gorbunov MY, Falkowski PG**. 2015a. An RNA interference knock-down of nitrate reductase enhances lipid biosynthesis in the diatom *Phaeodactylum tricornutum*. Plant Journal **84**, 963-973.

**Levitan O, Dinamarca J, Zelzion E, Lun DS, Guerra LT, Kim MK, Kim J, Van Mooy BAS, Bhattacharya D, Falkowski PG**. 2015b. Remodeling of intermediate metabolism in the diatom *Phaeodactylum tricornutum* under nitrogen stress. Proceedings of the National Academy of Sciences of the United States of America **112**, 412-417.

**Longworth J, Wu DY, Huete-Ortega M, Wright PC, Vaidyanathan S**. 2016. Proteome response of *Phaeodactylum tricornutum*, during lipid accumulation induced by nitrogen depletion. Algal Research - Biomass Biofuels and Bioproducts **18**, 213-224.

**Matthijs M, Fabris M, Broos S, Vyverman W, Goossens A**. 2016. Profiling of the early nitrogen stress response in the diatom *Phaeodactylum tricornutum* reveals a novel family of RING-domain transcription factors. Plant Physiology **170**, 489-498.

**Matthijs M, Fabris M, Obata T, Foubert I, Franco-Zorrilla JM, Solano R, Fernie AR, Vyverman W, Goossens A**. 2017. The transcription factor bZIP14 regulates the TCA cycle in the diatom *Phaeodactylum tricornutum*. Embo Journal **36**, 1559-1576.

**Popko J, Herrfurth C, Feussner K, Ischebeck T, Iven T, Haslam R, Hamilton M, Sayanova O, Napier J, Khozin-Goldberg I, Feussner I**. 2016. Metabolome analysis reveals betaine lipids as major source for triglyceride formation, and the accumulation of sedoheptulose during nitrogen-starvation of *Phaeodactylum tricornutum*. Plos One **11**.

**Provasoli L, McLaughlin JJA, Droop MR**. 1957. The development of artificial media for marine algae. Archiv fur Mikrobiologie **25**, 392-428.

**Remmers IM, D'Adamo S, Martens DE, de Vos RCH, Mumm R, America AHP, Cordewener JHG, Bakker LV, Peters SA, Wijffels RH, Lamers PP**. 2018. Orchestration of transcriptome, proteome and metabolome in the diatom *Phaeodactylum tricornutum* during nitrogen limitation. Algal Research **35**, 33-49.

**Remmers IM, Martens DE, Wijffels RH, Lamers PP**. 2017. Dynamics of triacylglycerol and EPA production in *Phaeodactylum tricornutum* under nitrogen starvation at different light intensities. Plos One **12**, e0175630.

**Roháček K, Soukupova J, Bartak M**. 2008. Chlorophyll fluorescence: A wonderful tool to study plant physiology and plant stress. In: Schoefs B, ed. *Plant Cell Organelles – Selected Topics*. Trivandrum: Research Signpost, 251-284.

**Smith SR, Dupont CL, McCarthy JK, Broddrick JT, Oborník M, Horák A, Füssy Z, Cihlář J, Kleessen S, Zheng H, McCrow JP, Hixson KK, Araújo WL, Nunes-Nesi A, Fernie A, Nikoloski Z, Palsson BO, Allen AE**. 2019. Evolution and regulation of nitrogen flux through compartmentalized metabolic networks in a marine diatom. Nature Communications **10**, article #4552.

**Valenzuela J, Mazurie A, Carlson RP, Gerlach R, Cooksey KE, Peyton BM, Fields MW**. 2012. Potential role of multiple carbon fixation pathways during lipid accumulation in *Phaeodactylum tricornutum*. Biotechnology for Biofuels **5**, 40.

**Walne PR**. 1966. Experiments in the large-scale culture of the larvae of *Ostrea edulis* L. Fishery Investigations **25**, 1-55.

**Wang X, Fosse HK, Li K, Chauton MS, Vadstein O, Reitan KI**. 2019. Influence of nitrogen limitation on lipid accumulation and EPA and DHA content in four marine microalgae for possible use in aquafeed. Frontiers in Marine Science **6**, article #95.

**Yang Z-K, Niu YF, Ma YH, Xue J, Zhang MH, Yang WD, Liu JS, Lu SH, Guan Y, Li HY**. 2013. Molecular and cellular mechanisms of neutral lipid accumulation in diatom following nitrogen deprivation. Biotechnology for Biofuels **6**, 67.

**Yodsuwan N, Sawayama S, Sirisansaneeyakul S**. 2017. Effect of nitrogen concentration on growth, lipid production and fatty acid profiles of the marine diatom *Phaeodactylum tricornutum*. Agriculture and Natural Resources **51**, 190-197.

**Zhao Y, Wang Y, Quigg A**. 2015. Comparison of population growth and photosynthetic apparatus changes in response to different nutrient status in a diatom and a coccolithophore. Journal of Phycology **51**, 872-884.
